# Recent progress in one dimensional TiO_2_ nanomaterials as photoanodes in dye-sensitized solar cells

**DOI:** 10.1039/d2na00437b

**Published:** 2022-09-30

**Authors:** Deepak Joshy, Soumya B. Narendranath, Yahya A. Ismail, Pradeepan Periyat

**Affiliations:** Department of Chemistry, University of Calicut Kerala 673635 India; Department of Chemistry, Central University of Kerala 671316 India; Department of Environmental Studies, Kannur University Kerala India pperiyat@kannuruniv.ac.in

## Abstract

Exploiting the vast possibilities of crystal and electronic structural modifications in TiO_2_ based nanomaterials creatively attracted the scientific community to various energy applications. A dye sensitised solar cell, which converts photons into electricity, is considered a viable solution for the generation of electricity. TiO_2_ nanomaterials were well accepted as photoanode materials in dye-sensitized solar cells, and possess non-toxicity, high surface area, high electron transport rates, fine tuneable band gap, high resistance to photo corrosion and optimum pore size for better diffusion of dye and electrolyte. This review focuses on various aspects of TiO_2_ nanomaterials as photoanodes in dye-sensitized solar cells. TiO_2_ photoanode modification *via* doping and morphological variations were discussed in detail. The impact of various morphologies on the design of TiO_2_ photoanodes was particularly stressed.

## Abstract 2

One-dimensional TiO_2_ nanomaterials as efficient DSSC photoanode materials.

## Graphical abstract

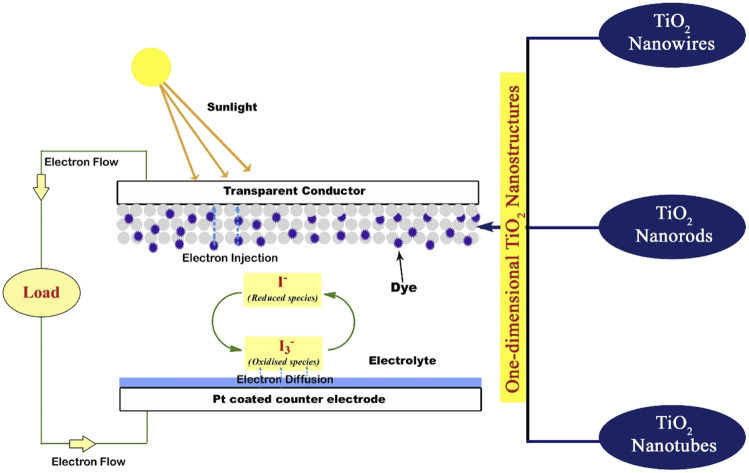

## 1. Introduction

The excessive consumption of fossil fuels to meet the increasing energy demand has resulted in profound consequences such as global warming, environmental pollution, *etc.* To address the challenges raised by environmental degradation and the energy crisis, the development of a sustainable energy production approach is required. Even though various renewable energy resources such as geothermal energy, biofuels, and wind-tidal energies can substantially contribute to sustainable energy development, solar energy has to play a major role. In this era of growing energy demand and environmental issues, solar energy is the only choice left in front of us as solar radiation possesses immense potential, vast abundance and environmental friendliness. Out of the 4 million exajoules (10^18^ J) of solar energy reaching the earth, around 50 000 EJ's are easily exploitable.^1^ The significance of solar energy becomes more clear from the fact that the world's annual energy consumption for the year 2017 was around 565 EJ's which is only a fraction of the harvestable solar energy (*i.e.* 50 000 EJ's).^2^ But in the present scenario, electricity produced from the renewable energy sector constitutes only 8.4% of the total world electricity production.^2^ More than 20% of the contribution to the renewable energy sector is made by solar energy.^3^ But this share of solar energy is significantly small compared to the magnitude of solar power available for harvesting. The lack of efficient and economically viable solar harvesting techniques is the main reason for this huge difference. Solar cells are one of the widely employed solar energy conversion devices. Solar cells are classified into different generations based on the material and technology present in them. We have first generation silicon solar cells as the most widely commercialized solar cell type.^4,5^ Silicon solar cells have efficiencies of around 26% which is almost near the theoretical efficiency maximum.^6^ At the same time, second-generation solar cells are based on thin-film technology and employ materials such as CdTe, CIGS, *etc.*^7–10^ The efficiency of second-generation solar cells has reached up to 21.7% recently.^6^ But the large-scale commercialization of first and second-generation solar cells is very expensive. Also, the components used in these two generations are capable of causing environmental hazards. So, to develop an economically viable and eco-friendly way of solar energy conversion, the third generation of solar cells, known as dye-sensitized solar cells (DSSCs) was introduced.^11–14^ The concept of a DSSC was introduced by Michael Gratzel in 1991.^15^ Significant attraction garnered by DSSCs is due to their large-scale and economically viable production capability along with flexibility. The efficiencies of DSSCs introduced so far are found to be within 14.3%.^16^ If the issues associated with low efficiency and shorter durability are addressed properly, DSSCs are the best to play a vital role in the development of a sustainable energy culture. Also, large-scale commercialization of DSSCs can convert a major share of the available solar radiation. Performance enhancement, durability increment and production cost reduction of DSSCs can be carried out in various ways. Mainly it involves either the synthesis of novel component materials/modification of existing materials or the introduction of more rational DSSC designs. As a DSSC assembly consists of various components such as a photoanode, sensitizer(dye), counter electrode, redox electrolyte, *etc.*, investigation into performance enhancement can be carried out on any one of these components. In this review, special emphasis is given to the DSSC photoanode and the contribution of one dimensional TiO_2_ nanomaterial based photoanodes for the critical development of DSSCs.

### 1.1. DSSC structure & working- an overview

**Fig. 1:**
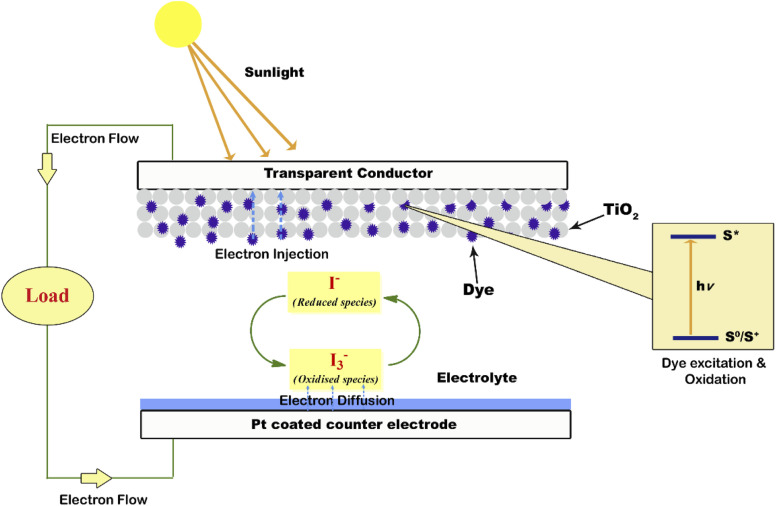
Schematic diagram of a DSSC.

A photoanode forms the core part of the DSSC and it consists of a transparent conducting oxide (TCO) glass plate or plastic substrate on which nanometre-sized semiconducting metal oxide particles are deposited and sintered. Fluorine-doped tin oxide (FTO) is the most widely employed substrate.^17,18^ Usually, a mesoporous TiO_2_ film having a thickness of around 10 µm is coated on the FTO substrate to facilitate electron transfer.^19^ Sensitizer or dye molecules responsible for light absorption are adsorbed onto the mesoporous nanomaterial film. Upon irradiation by solar radiation, the dye molecule will get excited and emit an electron. This emitted electron will get injected into the conduction band of the nanocrystalline semiconductor metal oxide film. From there the electron will get transferred to the FTO plate and then to the external circuit. The dye molecule which got oxidised on solar irradiation is now regenerated by employing a redox couple.^20,21^ The iodide/triiodide redox system is the most widely used one.^22^ Upon reduction of the oxidised dye molecule, the iodide ion gets oxidised into triiodide. The regeneration of the redox couple is facilitated by electrons which reach the counter electrode through the external circuit. The counter electrode consists of a thin layer of Pt nanoparticles deposited on a conducting glass substrate.^23,24^ Here the electron emitted from the dye molecule travels through the mesoporous nanomaterial layer deposited on the FTO plate and then flows through the external load and finally reaches the counter electrode to regenerate the redox system. The redox electrolyte system present in between the two electrodes not only facilitates dye regeneration but also prevents the recombination of conduction band electrons with oxidised dye molecules. While fabricating the DSSC, as shown in Fig. 1 the photoanode and cathode are sandwiched together with a layer of the polymer film in between them.^25^ Then the redox electrolyte system is injected into the space in between so that the triiodide ions can diffuse towards the counter electrode and get reduced to iodide ions. A number of such repeating cycles produce current in the circuit. The resultant efficiency of a DSSC depends on the electron recombination rate. The lower the electron recombination rate, the higher the efficiency will be. The lower electron recombination rate results from the high electron transport rate.^26^

### 1.2. Important terms associated with DSSCs

The important parameters associated with dye sensitised solar cells are incident photon-to-current efficiency (IPCE), open circuit voltage (*V*_OC_), short circuit current (*J*_sc_), fill factor (FF) and cell efficiency.

#### 1.2.1 Short circuit photocurrent (*J*_sc_)

**Fig. 2:**
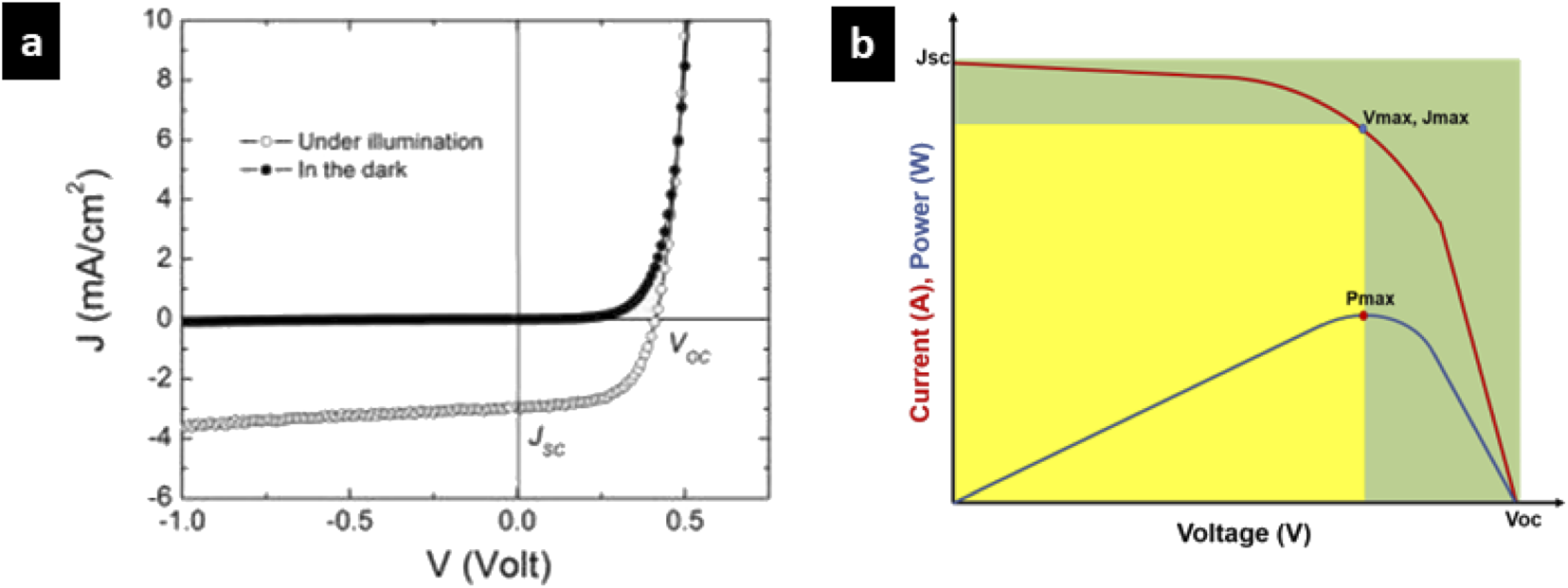
(a) The current *versus* voltage curve associated with a solar cell circuit in the dark and under illumination (Copyright: https://depts.washington.edu/). (b) Graphical representation of the fill factor.

Fig. 2a shows the current *versus* voltage curve associated with a solar cell circuit in the dark and under illumination. When the electrons travel from the anode to the cathode of the solar cell, and on the other hand when the circuit is reverse biased, the current flows will be zero. This is due to the hindrance caused by the high energy barrier of the donor. After the energy barrier is crossed, a very low current will flow. This feature is represented by the dark curve. When the solar radiation is absorbed by the donor, charge carriers will be generated easily. There will be a reverse current due to the electron flow from the anode to cathode. This reverse current which has resulted in the absence of an external voltage is termed as photocurrent or short circuit photocurrent (*J*_sc_). This is represented by the hollow bubbled curve.

#### 1.2.2 Open circuit voltage (*V*_OC_)

Since the short circuit current is a result of the reverse bias current, it is possible to compensate the current by applying a forward voltage. Under this condition, there will be a point where the current becomes zero. The voltage corresponding to this point is referred to as the open circuit voltage (*V*oc). On the other hand, the open circuit voltage is the maximum voltage available from a solar cell when there is no external load connected and the external current flowing through the cell is zero.^27^

#### 1.2.3 Fill factor (FF)

The solar cell fill factor (FF) gives us an idea of the performance of the solar cell.^28^ It corresponds to the ratio of the actual performance to the theoretical maximum power output of a solar cell.
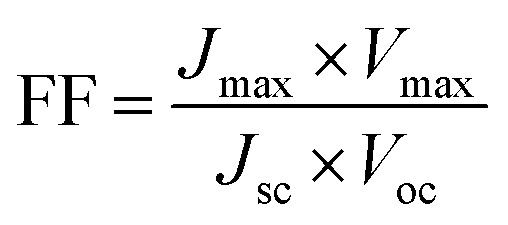


A higher fill factor favours the maximum output of a solar cell. The graphical representation of the fill factor is shown in Fig. 2b. This graph shows the solar cell output current and power as a function of voltage. It is the ratio of the area of the larger rectangle (pale green) to the area of the smaller rectangle (yellow).

#### 1.2.4 Incident photon-to-current efficiency (IPCE)

The incident photon-to-current efficiency (IPCE) is a measure of the ratio of the photocurrent (converted to an electron transfer rate) *vs.* the rate of incident photons (converted from the calibrated power of a light source) as a function of wavelength.^29^ IPCE is also known as quantum efficiency which measures the efficiency of a device to convert incident photons into electrical energy at a given wavelength.

#### 1.2.5 Solar cell efficiency

The efficiency of a solar cell is defined as the fraction of incident solar power which is converted to electricity. Solar cell efficiency depends on the spectrum, intensity of incident sunlight and the temperature of the solar cell. Therefore, conditions under which efficiency is measured must be carefully controlled to compare the performance of one device to that of another one.^30^ Terrestrial solar cells are measured under AM1.5 conditions and at a temperature of 25 °C. The maximum output power is given by*P*_max_ = *V*_oc_ × *J*_sc_ × FFThen efficiency is given by the equation,*η* = *V*_oc_ × *J*_sc_ × *FF*/*P*_in_where *V*_OC_ is the open circuit voltage, *J*_SC_ is the short circuit current and FF is the fill factor.^11^

### 1.3. Role of the photoanode in a DSSC

Among the functional components of a DSSC, the photoanode forms the crucial part. The nature of the material used as the photoanode and its morphology are important factors determining the overall performance of a DSSC. The functions of a photoanode include dye pickup, electron injection, transportation and collection which in turn influence the photocurrent, photovoltage and power conversion efficiency. Efforts are being made to improve the efficiency of DSSCs by introducing novel materials as photoanodes and by modifying the morphologies of existing materials. The essential requirements for an ideal photoanode are:

(i) Higher surface area facilitates the adsorption of dye molecules to a greater extent.^31,32^

(ii) Photoanodes should have higher electron transport rates so that the electron injection from the dye to the external circuit through the photoanode occurs smoothly.^33^

(iii) Photoanodes should have suitable band gap alignment with the energy levels of the sensitizer.^34^

(iv) It should possess high resistance to photo corrosion.^35^

(v) Photoanode materials should possess a pore size that can be optimized to achieve better diffusion of dye and electrolyte.^36^

(vi) It should possess the ability to absorb/scatter sunlight for the improved performance of the dye.^37^

(vii) The photoanode material needs to be in optimum contact with the dye molecules and the conducting substrate.^38^

The above-mentioned characteristics are vital in achieving a better photoconversion efficiency.

This review discusses the significance of TiO_2_ nanomaterials as photoanodes in DSSCs. The characteristic photovoltaic properties and important modifications of TiO_2_ for photoanode applications are surveyed. A summary of research on TiO_2_ and its important one-dimensional morphologies and their modifications to be used as photoanode materials in DSSCs is given here.

## 2. Photoanode materials

**Fig. 3:**
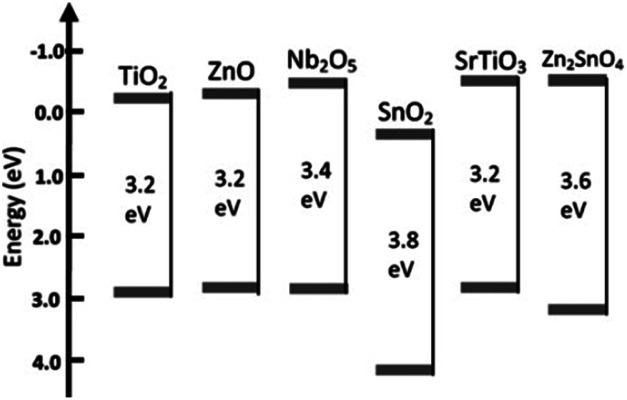
Band positions of various semiconductor oxide materials used as photoanodes.^57^ (Reprinted with permission from ref. Yang *et al.*, 2013. Copyright 2013 WILEY‐VCH Verlag GmbH & Co. KGaA, Weinheim.)

The limitations associated with the conventional materials lead to the investigation of more effective photoanode materials, which have advanced properties with bulk and surface modifications. Commonly investigated materials include metal oxides such as TiO_2_, ZnO,^39–42^ Nb_2_O_5_,^43–45^ SnO_2_,^46–48^ SrTiO_3_,^49,50^ WO_3_,^51–53^ and Zn_2_SnO_4_.^54–56^ All these are wide band gap semiconductor materials whose structure, morphology and crystallinity decide the performance of the DSSCs. The band structures of these materials are shown in Fig. 3.^57^

**Fig. 4:**
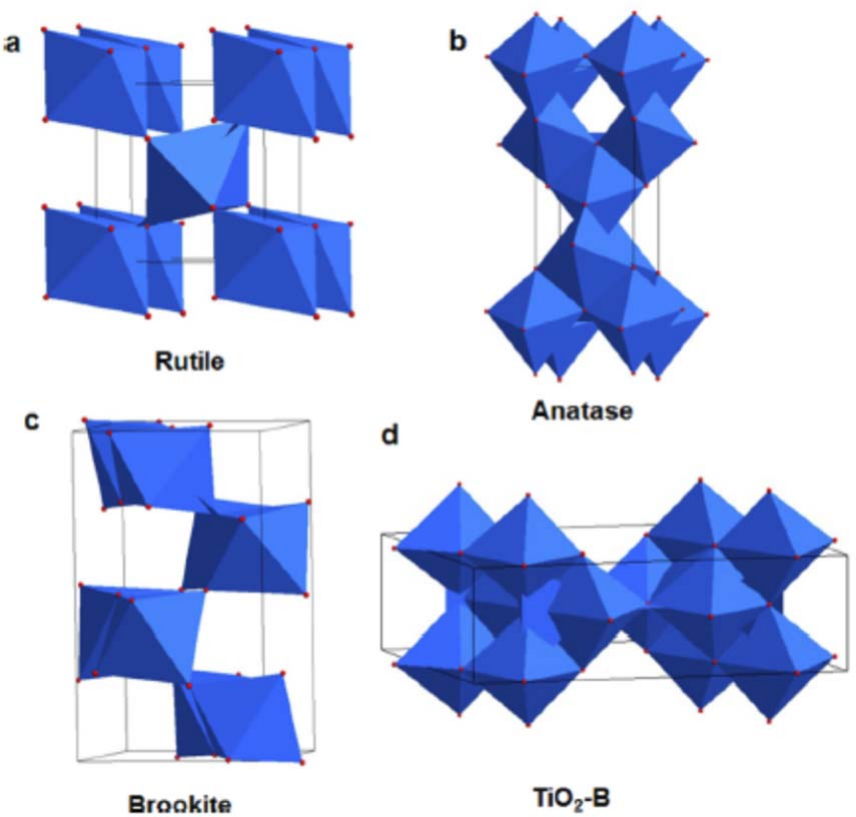
Schematic unit cell structure of four TiO_2_ polymorphs: (a) rutile (b) anatase (c) brookite and (d) TiO_2_ (B)^66^ (Reprinted with permission from ref. Wang, X. *et al.*, 2014. Copyright 2014 American Chemical Society).

Among these materials, TiO_2_ and ZnO are the most widely used materials for DSSC fabrication.^58–62^ Although ZnO has better electron mobility compared to TiO_2_, its efficiency is less than those DSSCs employing TiO_2_. This lower efficiency of ZnO emerges due to decreased dye adsorption and instability in acidic environments.^63^ Therefore, TiO_2_ has superior photovoltaic applicability compared to ZnO. Four polymorphs of TiO_2_ are known, *viz*, as rutile (tetragonal), anatase (tetragonal), brookite (orthorhombic) and TiO_2_ (B) (monoclinic) as shown in Fig. 4.^64–66^ Among these polymorphs of TiO_2_, anatase is preferred over rutile for photovoltaic applications irrespective of rutile's greater stability and lower band gap. This is because the anatase phase has a higher conduction band energy level, absorptive affinity and a lower electron–hole recombination rate.^67^ Since the synthesis of brookite TiO_2_ is difficult, its applicability as a photoanode remains less explored.^68^

**Fig. 5:**
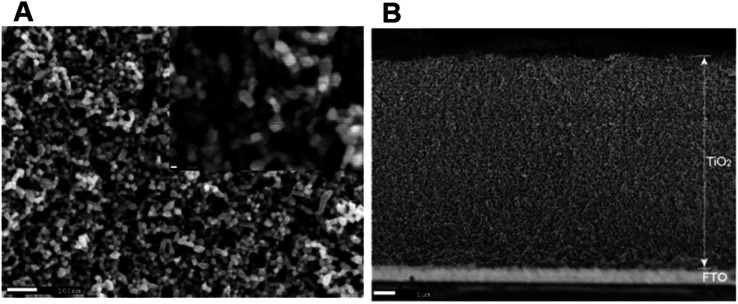
(a) SEM images of mesoporous TiO_2_ and (b) film thickness of mesoporous TiO_2_^73^ (Reprinted with permission from ref. Muniz *et al.*, 2011. Copyright 2010 Elsevier).

**Fig. 6:**
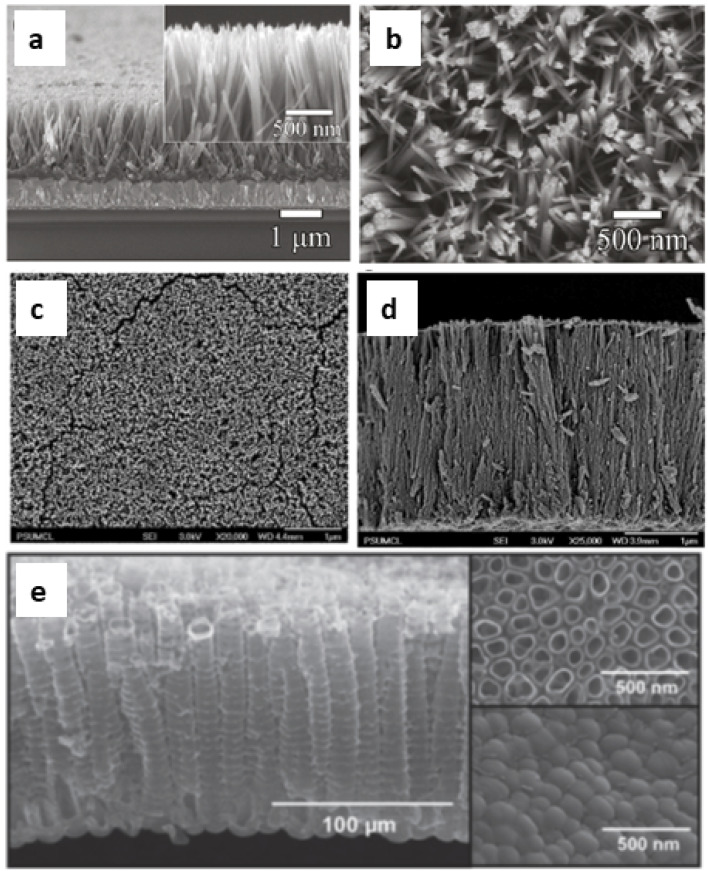
SEM images: (a) cross sectional view and (b) top view of TiO_2_ nanorods^74^ (Reprinted with permission from ref. Cho *et al.*, 2011. Copyright 2011 American Chemical Society); FESEM images (c) top view images and (d) cross sectional image of a TiO_2_ nanowire array on FTO coated glass^75^ (Reprinted with permission from ref. Feng *et al.*, 2008. Copyright 2008 American Chemical Society) and (e) SEM images of TiO_2_ nanotubes^76^ (Reprinted with permission from ref. Roy *et al.*, 2010. Copyright 2009 Royal Society of Chemistry).

DSSCs employing anatase TiO_2_ showed efficiencies ranging from 12–14%.^69,70^ So TiO_2_ is considered the best choice available as a photoanode material due to its (a) cost-effectiveness, (b) easy availability, (c) good stability along with non-toxicity and (d) suitable optical and electronic characteristics.^67^ Furthermore, most of the stable sensitizers showing higher light absorption capability have their LUMO positioned favourably with the conduction band of TiO_2_. These favourable qualities of TiO_2_ initiated further investigations to improve the functioning of TiO_2_ photoanodes. During this process of improvement, the main challenges to deal with include the (i) large band gap (3.2 eV) of TiO_2_ causing adsorption in the UV region^71^ and (ii) low internal electron transport rate.^72^ Furthermore, research aimed at better performance of TiO_2_ by focusing on factors such as high surface area, an increased light scattering effect, enhanced interface quality, fast electron mobility and better charge collection ability. The physical, chemical and optical properties of TiO_2_ depend not only on its intrinsic electronic structure but also on its shape, size, porosity, pore size distribution, organization and surface features. One approach to increase the photoconversion efficiency is by maximising the surface area of TiO_2_ and thereby enhancing the reaction at the interface of the photoanode and interacting media. The extent of dye adsorption depends on the surface area available. The greater the dye pickup, the more the electron/current density that will be generated. Semiconductor mesoporous TiO_2_ (Fig. 5), nanorods (Fig. 6a and b), nanowires (Fig. 6c and d), nanotubes (Fig. 6e), nanosheets and various other nanoarchitectures have been employed and explored for enhanced dye adsorption.^73–76^ Besides increasing the surface area, enhancing electron mobility is also a crucial factor in improving the performance of the photoanode. Defects in TiO_2_ act as electron traps at the grain boundaries and the absence of defects will assure better collection of injected electrons from the semiconductor. Another method involves surface modification of TiO_2_ semiconductors which has remarkable influence on charge separation, electron mobility and the recombination process.^77^ Attempts are being made to minimise electron recombination losses due to grain boundaries and also to extend the absorption of TiO_2_ towards the near-infrared region.

### 2.1. TiO_2_ photoanode modification by doping

Electronic properties of TiO_2_ can be effectively modified by doping *i.e.* by the deliberate insertion of impurities into the TiO_2_ lattice.^78^ Doping in the TiO_2_ lattice results in an increase in free charge carriers and conductivity.^79^ This is due to the defect-ridden nature of TiO_2_ and it influences the electronic structure and trap states present in the lattice.^80^ While doping, either the Ti^4+^ cation or O^2−^ anion can be replaced. TiO_2_ has a band structure consisting of conduction band (CB) energy levels formed by the Ti^4+^ orbitals and valence band (VB) energy levels comprised of O^2−^ 2p orbitals.^67^ Thus replacing Ti^4+^ and O^2−^ ions will alter the CB and VB structures respectively.^81^ The atomic radii of the dopants should be comparable to the ions to be replaced.^82^ The sensitizer dye molecules used to bind with Ti atoms in the TiO_2_ lattice and the replacement of Ti with other atoms will affect the dye adsorption due to different binding strengths between the dye and the dopant.^83,84^ It was also observed that the growth rate of TiO_2_ nanoparticles is inhibited by dopants resulting in smaller particles having increased surface area.^85,86^ As a result of an increase in surface area, dye adsorption and current densities are improved.^87^ This will automatically enhance light absorption and facilitate the use of thinner films having a lower recombination rate in DSSCs.^32^

Various morphologies of TiO_2_ have a major influence on its optical and electronic properties.^88^ It is found that one-dimensional nanostructures such as nanowires, nanorods and nanotubes possess better charge transport properties compared to nanoparticle assemblies.^89^ However, these 1D structures have lesser surface area than nanoparticle systems and thereby have reduced dye pick-up capability.^90^ Dopants having better dye adsorption capacities can improve the photovoltaic performance of 1D nanostructures.^91,92^ At the same time, nanoparticle assemblies benefit from dopants that cause increased charge transfer.^93^ Hence, it is difficult to identify clearly whether performance improvement is caused by an increase in absorption or electronic effects in doped TiO_2_.

Based on the general electronic configuration, dopants can be classified into alkaline earth metals,^94–96^ metalloids,^97–99^ non-metals,^100–102^ transition metals,^103^ post-transition metals^104,105^ and lanthanides.^106–108^ Significant attempts were made for co-doping in which more than one dopant is introduced into the lattice for enhanced device performance.^109–111^ Doping in a TiO_2_ lattice alters its phase, flat band potential, recombination rate, electron transport rate and dye adsorption capability.^79^ Anatase to rutile phase transformation is inhibited by doping,^112^ thereby reducing charge carrier recombination. The flat band potential will experience either a positive or a negative shift *i.e.* a positive shift involves the downward shift of the CB and Fermi level (*E*_F_) whereas a negative shift involves the upward shift of the CB and *E*_F_. A decrease in the number of defect states upon doping increases the lifetime of photogenerated electrons and reduces recombination losses.^113^ At the same time, a decrease in the number of trap states may lead to enhanced electron mobility.^114^ Dopants influence the growth rate of nanoparticles, thereby affecting their size, surface area and the number of grain boundaries. Also, dopants decide the binding strength between the doped surface and dye molecules.^115^ Recently, Mn-doped TiO_2_ with IR absorbing capability was reported to be an excellent photoanode for dye-sensitized solar cells and it possesses 79% higher efficiency compared to commercial P25.^116^

### 2.2. Nanostructured TiO_2_ photoanode

A revolutionary breakthrough in the field of photoanode fabrication with the introduction of a mesoporous TiO_2_ nanoparticle photoanode was pioneered by Gratzel and co-workers in 1991.^117^ They replaced the bulk TiO_2_ photoelectrode with a nanostructured architecture and obtained an efficiency of 7.1–7.9% by employing a trimeric Ru complex.^118^ Detailed guidelines for the fabrication of a TiO_2_ nanoelectrode for DSSCs with efficiency >10% are given by Gratzel and team.^119^ They introduced a double-layered TiO_2_ film comprising of a light-absorbing layer of anatase with 20 nm thickness and a light scattering overlayer of 200–400 nm sized anatase particles.^119^ Soon several multi-layered TiO_2_ photoanodes were introduced to achieve broadband light confinement without affecting dye adsorption capacity.^120^

Several modifications and novel nanoarchitectures of TiO_2_ have been introduced to exploit the nanoscale properties for better DSSC performance.^121^ Zero dimensional TiO_2_ nanoparticles serve as the growth centres for advanced nanoarchitectures with enhanced performance parameters. When the particle size approaches the nanometre range, the nanostructure band gap increases as a result of quantum size effects and it is possible to adjust the valence and conduction band energy levels of the nanosemiconductor with respect to the redox potentials of the redox couple used.

## 3. One-dimensional nanostructures as photoanodes

One-dimensional nanostructures employed in DSSCs consist of nanotubes, nanowires, nanofibres, nanobelts and nanoribbons. One-dimensional nanomaterials gained attention through the pioneering work by Iijima.^122^ A well-ordered arrangement of one-dimensional nanostructures can provide a direct electron transport pathway from the semiconductor film to the conducting substrate. This results in a reduction in the electron recombination rate and an increase in PCE.^123^

### 3.1. Nanowires

**Scheme 1:**
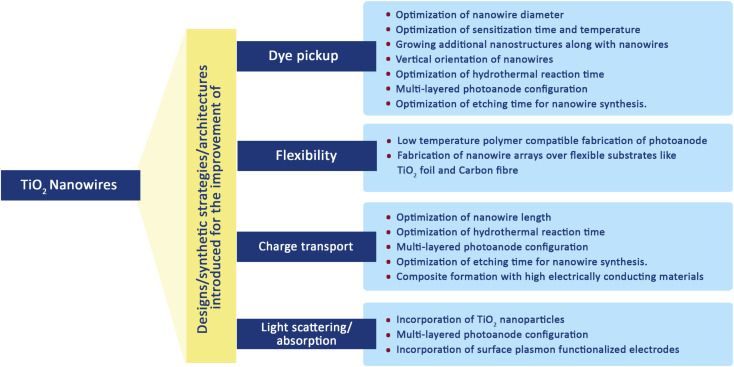
Various modifications introduced for the improvement of TiO_2_ nanowire based DSSC performance.

TiO_2_ nanowire structures serve as confined conducting channels with their long charge diffusion lengths preventing charge recombination and thus facilitating better charge transport.^66^ It is this fast charge transport and better charge collection ability which made them possible candidates for DSSC fabrication. By using a dense array of long, thin nanowires as dye scaffolds, it is possible to increase both dye pickup and carrier collection efficiency. Also, nanowire photoanodes are found to be more suitable for non-standard electrolytes such as solid inorganic phases or polymer gels having higher recombination rates.^123^ Transient photocurrent and photovoltage measurements were carried out on TiO_2_ nanowires and it was found that the electron transport time and its dependence on illumination intensity are similar to that of TiO_2_ nanoparticles.^124^ However, the ratio of electron–hole recombination time and electron collection time of TiO_2_ nanowire-based DSSCs are about 150 times that of nanoparticle-based solar cells and it indicates the improved collection efficiency of nanowire arrays. Scheme 1 gives a brief idea of various attempts made to improve the key factors affecting the overall performance of TiO_2_ nanowire based DSSCs. From the large collection of literature on TiO_2_ nanowire based DSSCs, here we are selectively discussing major research outcomes involving efficient synthesis methods for nanowires, novel architectures and significantly enhanced efficiencies. Many of the nanowire synthesis methods were based on hydrothermal treatment with slight modifications.

**Fig. 7:**
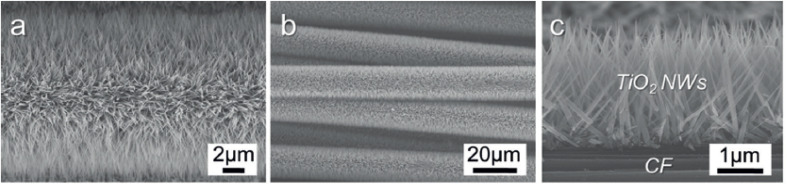
(a–c) Field emission (FE)-SEM images of TNW arrays grown on a CF substrate at 190 °C for 80 min.^126^ (Reprinted with permission from ref. Cai *et al.*, 2014. Copyright 2014 WILEY-VCH Verlag GmbH & Co. KGaA, Weinheim.)

Feng *et al.* presented a straightforward low-temperature method to fabricate single-crystalline rutile TiO_2_ nanowires through a nonpolar solvent/hydrophilic solid substrate interfacial reaction under hydrothermal conditions.^75^ Nanowires having lengths up to 5 µm can be grown vertically from the TCO glass substrate along the preferred direction by this approach. This arrangement has given an efficiency of 5.02% under AM1.5 irradiation by employing N719 dye on 2–3 µm long TiO_2_ nanowire arrays. An added advantage of this technique is the low temperature employed, which favours the use of polymers for cell fabrication. Thus, low-temperature methods of photoanode fabrication are found to be compatible with polymer substrates and can result in flexibility. As part of developing flexible and lightweight DSSCs, Liao *et al.* introduced hierarchial TiO_2_ nanowire (HNW) arrays grown on a Ti foil substrate instead of FTO.^125^ These HNW arrays contain long TiO_2_ nanowire trunks and short TiO_2_ nanorod branches and are prepared by a two-step hydrothermal process. Liao *et al.* are the first to report such HNW arrays fabricated on Ti foil and they also replaced the Pt counter electrode with another electrode in which PEDOT is electrodeposited on the ITO-PET substrate. Even though HNW array DSSCs showed increased efficiency (4.32%) compared to that of NW-based DSSCs, the former possesses a comparatively reduced electron lifetime and transport time. As a further step toward realising lightweight and flexible DSSCs, cells were constructed with a vertical TiO_2_ nanowire array grown *in situ* on carbon fibre substrates as shown in Fig. 7.^126^

**Fig. 8:**
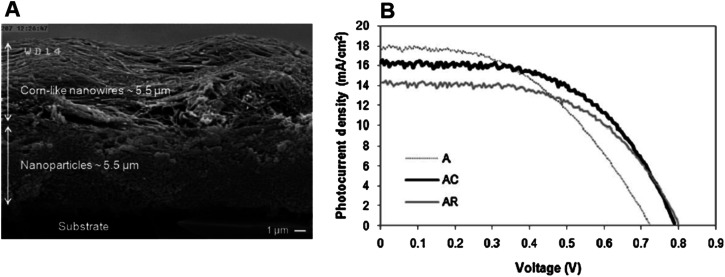
(a) FE-SEM image of the cross sectional view of a double-layer film containing TiO_2_ nanoparticles as the under-layer and corn-like TiO_2_ nanowires as the over-layer. (b) Photocurrent density–voltage curves of fabricated TiO_2_ solar cells [A-TiO_2_ NP solar cell, AC- Corn like TiO_2_ NW solar cell, AR-Regular TiO_2_ NW solar cell].^129^ (Reprinted with permission from ref. Bakhshayesh, A. *et al.*, 2013. Copyright 2012 Elsevier Ltd.)

In 2015, Liu *et al.* reported the controllable formation of TiO_2_ nanowire arrays on a titanium mesh. This method is a hydrothermal one capable of forming nanowire arrays (NWAs) with an average diameter of 80 nm.^127^ Along with studying the influence of NWA preparation conditions on DSSC parameters, Liu *et al.* also focused on the role of the sensitization temperature and time on DSSC performance.^127^ They found that a higher sensitization temperature would benefit dye molecule infiltration to the internal areas of NWA films, and the complete covering of monolayer dye molecules on the surface of TiO_2_ NWAs would enhance the photovoltaic properties of the DSSC. By maintaining optimum conditions, Liu *et al.* obtained an efficiency of 3.42% for a flexible DSSC. Apart from maintaining optimum dye pickup, they were found to have depressed charge recombination also. Later several groups tried enhancing the available surface area of the photoanode by growing additional structures on TiO_2_ nanowires. In 2014, single-crystal-like 3D TiO_2_ branched nanowire arrays were fabricated by Sheng *et al.* with the 1D branch epitaxially grown from the primary trunk.^128^ This attempt made by Sheng *et al.* increased the available surface area by 71% and they also exhibited a fast charge transport property compared to one-dimensional TiO_2_ nanowires. Sheng obtained an efficiency of 4.61% for branched nanowire-based DSSCs.^128^ It is found that the presence of other nanostructures as branches on nanowires creates additional boundaries in electron transport. Later, DSSCs with multilayered photoanodes, in which different functions were assigned to specific layers, were fabricated. For example, in 2013, Bakshayesh *et al.* introduced a new TiO_2_ structure with corn-like nanowire morphology having high surface area and crystallinity synthesised by the surface tension stress mechanism.^129^ They have adopted a double-layer DSSC design consisting of an underlayer of anatase TiO_2_ nanoparticles and an overlayer of corn-like TiO_2_ nanowires as shown in Fig. 8a and Fig. 8b shows the photocurrent against voltage curves. By adopting a triple function mechanism with effective management of light scattering, dye sensitization and photogeneration of charge carriers, Bakshayesh *et al.* attained an efficiency of 7.11%. Here the increased surface area of corn-like nanowires resulted in improved dye sensitization and short circuit current density. At the same time, the presence of NPs on corn-like nanowires increased light scattering. Most of the multilayer photoanodes follow such division of labour among various layers. Another new morphology reported in 2013 consists of thornbush like TiO_2_ nanowires (TBWs) prepared by a facile single-step hydrothermal method using potassium titanium oxide oxalate dehydrate, diethylene glycol (DEG) and water at 200 °C.^130^ These TBWs consist of a large number of nanoplates and nanorods. Depending on the change in the DEG/water composition, the diameter, as well as the morphology of TBWs, varies. TBWs having a diameter of 200 nm shows higher efficiency (5.2%) than those having a diameter of 400 nm (4.5%) and 600 nm (3.4%). Further treatment of TBW200 with graft-copolymer-directed, organized mesoporous TiO_2_ helps to increase the surface area and interconnectivity of TBWs leading to an enhanced efficiency of 6.7%. As the electron transport in TiO_2_ nanowires depends on their length, efforts were made to modify the nanowire length. In 2011, a multicycle hydrothermal synthetic process to produce vertically oriented, single-crystalline rutile TiO_2_ nanowires with lengths in between 1 and 8 µm was reported by Zhou and co-workers.^131^ It is observed that a further increase in the nanowire length does not give an expected increase in efficiency. This can be attributed to a decrease in surface area by fusion and widening at the base of nanowires.

**Fig. 9:**
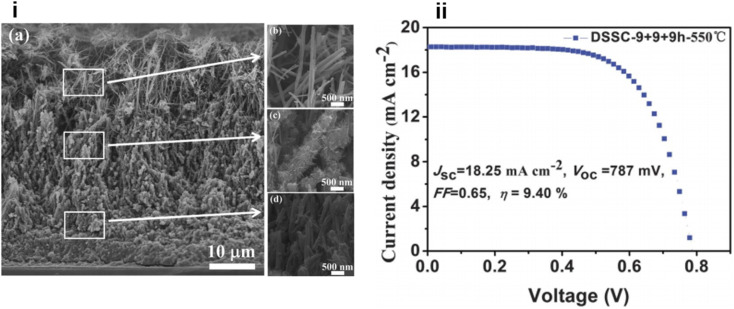
(i) (a) The cross-sectional SEM images of MTNWs on FTO glass prepared after three hydrothermal cycles (9 + 9 + 9 h); (b, c, and d) the FE-SEM images of the upper, intermediate and bottom layers of the as prepared MTNWs, respectively. (ii) *J*–*V* characteristic of DSSCs based on the 47 mm thick TiO_2_ NWs prepared with 3 hydrothermal cycles and followed by 550 °C calcination treatment.^134^ (Reprinted with permission from ref. Wu *et al.*, 2014. Copyright 2013 Royal Society of Chemistry.)

In the same year, a double-sided brush-shaped (DSBS) TiO_2_ nano architecture consisting of highly ordered TiO_2_ nanowires aligned around an annealed TiO_2_ nanoparticle layer was prepared by C. Zha and team *via* the hydrothermal method.^132^ Here nanowire growth is seeded by the annealed nanoparticle layer and it supports the DSBS structure. It was found that by varying the hydrothermal reaction time, structural properties like the crystalline phase, phase composition, length of the nanowires and thickness of the nanoparticle layer can be tuned. They have obtained an efficiency of 5.61% from an 8 hour reaction time and nanowires of length 6 µm. Thus, the microstructure has a significant influence on solar cell performance and the DSBS structure is a greatly promising one. Later Qiang Wu and co-workers fabricated a DSSC based on a self-assembled, vertically aligned TiO_2_ nanowire photoelectrode sensitized with N719 (ref. 133) sensitizer and it could achieve an efficiency of 9.40% (Fig. 9).^134^ Here TiO_2_ nanowires on an FTO glass plate have a tuneable length in the range of 15–55 µm and are suitable for multi-layered photoanode configuration.^134^ The same team of Wu *et al.* reported another distinct and novel architecture with the integration of various three-dimensional, hyperbranched titania nanoarchitectures into a multi-stack. The multi-stacked layers consist of an underlayer of hyperbranched hierarchical tree-like titania nanowires, branched hierarchical rambutan-like titania hollow submicrometer-sized spheres as the intermediate layer and hyperbranched hierarchical urchin-like titania micrometre-sized spheres as the top layer.^135^ Here the bottom layer enhances electron transport through one-dimensional nanowires into the FTO plate whereas the middle layer can guarantee effective light-trapping efficiency through the hollow-hole structure of submicrometer-sized macroporous TiO_2_ spheres and the upper layer of hyperbranched hierarchical TiO_2_ microspheres can offer increased light scattering ability. This integrated photoanode achieved an efficiency of 11.01% which is far better compared to that of its TiO_2_ NP analogue.

In 2016, Hailiang Li *et al.* constructed vertically aligned rutile TiO_2_ nanowire arrays (NWAs) with a length of ∼44 µm on transparent conductive fluorine-doped tin oxide (FTO) glass by a facile one-step solvothermal method without using any surfactant or template.^136^ By controlling the ethanol content in the reaction mixture, the length and separation between the NWs can be controlled. A reaction mixture containing 20 mL ethanol facilitated the formation of TiO_2_ NWAs with an efficiency of 8.9%. This can be further enhanced to 9.6–10.2% by incorporating a light scattering layer into the TiO_2_ NWA-based DSSC.

Another mode of enhancement of light absorption and efficiency of DSSCs utilizes the anti-reflecting (AR) property of photoanode materials. Another approach is by the exploitation of the surface plasmonic effect of metal nanoparticles. Yen *et al.* fabricated a DSSC utilizing both the antireflecting character and plasmonic effect. Their DSSC was equipped with a 3D TNW-AuNP plasmonic electrode having antireflective (AR) TiO_2_ nanowires (TNWs) serving as light-harvesting antennae coupled with Au nanoparticles (NPs).^137^ These plasmonic functionalized electrodes (PFEs) exhibited a remarkable plasmonic red shift from 520 nm to 575 nm. Such PFEs were developed to overcome the narrow absorption range and low absorption coefficient of dyes. It was found that TiCl_4_ treatment can increase the efficiency of TNW-AuNP hybrid DSSCs from 6.25% to 9.73%. In the same year, Lee *et al.* investigated the photovoltaic performances of back-illuminated DSSCs employing TiO_2_ NP/NW composite films of various weight percentages.^138^ These DSSCs used a cobalt-based electrolyte system. In NP/NW composite films NPs help to increase the surface area whereas NWs facilitate efficient charge transfer. The highest efficiency of 7.37% was shown by DSSCs with 10 wt% of NWs in the NP/NW composite film which is 20% improved compared to that of a pure NP-based DSSC. Apart from the widely used hydrothermal method of nanowire synthesis, Jin *et al.* proposed an approach for fabricating TiO_2_ nanowire networks on Ti foil using a Ti corrosion reaction in KOH aqueous solutions at different temperatures, followed by a further ion-exchange process.^139^ These photoanodes were suitable for bendable DSSCs and have exhibited an efficiency of 1.11% for back illumination. The same team also tried growing TiO_2_ nanowire networks on FTO substrates by wet corrosion and obtained an efficiency of 1.0% under AM 1.5 illumination.^140^ In 2017 Li and co-workers reported a simple single-step hydrothermal method to prepare single-crystalline self-branched anatase TiO_2_ NWs by using TBAH and CTAB as co-surfactants.^141^ These single crystalline self-branched TiO_2_ NW-based DSSCs exhibited an efficiency of 6.37% which is due to an enhanced percentage of exposed (010) facets having high dye adsorption capacity. Again in 2018, another design was introduced into the class of FDSSCs (fiber shaped dye-sensitized solar cells) which consists of a stretchable spring-like Ti@TiO_2_ nanowire array as the photoanode by Liu and co-workers. This design was intended to achieve higher flexibility and elasticity of DSSCs and the spring-like Ti@TiO_2_ array was prepared by the hydrothermal technique at a controlled NaOH concentration. Liu's team was the first to achieve a 100% stretching degree in FDSSCs with a PCE retention rate of 95.95% upon bending accompanied by 100% stretching strain.^142^ Another major finding was made by Ni *et al.*, that the enhanced surface area of nanowire arrays achieved on prolonged etching treatment can improve the dye intake but it reduces the PCE by increased electron recombination. Even then, Ni and co-workers succeeded in attaining an improved PCE of 9.39% by employing an additional scattering layer of TiO_2_ particles along with rough surface rutile TiO_2_ nanowire arrays.^143^ Thus, light scattering layers can make up for the loss encountered by extended rates of photo electron recombination. As a way of minimising charge recombination and improving electron transport across TiO_2_ nanowires, developing composites with materials having high electrical conductivity and charge carrier mobility was found exciting. One such approach was carried out by Makal and Das in 2021 by fabricating a reduced graphene oxide laminated one-dimensional TiO_2_–B nanowire composite based photoanode.^144^ The effect of different reduced graphene oxide loadings on the PCE of the DSSCs was evaluated and it was found that an 8 wt% loading showed a PCE of 4.95%. Makal and Das used the same design to develop another photoanode material where they encapsulated TiO_2_–B nanowires with graphitic carbon nitride (g-C_3_N_4_) and the PCE for the developed cell was found to be 5.12%.^145^ This is one of the highest efficiencies reported for a DSSC with a TiO_2_–B based photoanode. Significant efforts carried out for increasing the performance of nanowire-based DSSCs are discussed above and are tabulated in Table 1.

**Table 1:** TiO_2_ nanowire-based DSSCs

DSSC Type	Author	Photoelectrode and method of preparation	Sensitizer	Electrolyte	Efficiency	Ref.
DSSC with TiO_2_ nanowire arrays	Feng *et al.*	Vertically aligned single-crystalline TiO_2_ nanowire arrays by nonpolar solvent/hydrophilic solid substrate interfacial reaction under hydrothermal conditions	N719	MPN-100 (Solaronix, Inc., Switzerland) containing tri-iodide in methoxypropionitrile	5.02%	75
DSSC with hierarchical TiO_2_ nanowires (HNW)	Liao *et al.*	Hierarchical TiO_2_ nanowire (HNW) arrays grown on a Ti foil substrate instead of FTO by a two step hydrothermal process	N719	I^−^/I_3_^−^ based liquid electrolyte, which contained 1-methyl-3-propylimidazolium iodide (PMII), guanidinium thiocyanate and *tert*-butylpyridine in acetonitrile and valeronitrile	4.32%	125
DSSC with a TiO_2_ nanowire array grown *in situ* on carbon fibre	Cai *et al.*	Vertically aligned TNW arrays *in situ* grown on carbon fiber (CF) substrates through a facile, controllable, and seed-assisted thermal process	N719	I_2_, 1,3-dimethylimidazolium iodide, 4-*tert*-butylpyridine, guanidine thiocyanate, and LiClO_4_ in acetonitrile	4.18%	126
DSSC with TiO_2_ nanowire arrays on a titanium mesh	Liu *et al.*	Controllable formation of TiO_2_ nanowire arrays with average diameter 80 nm on a titanium mesh by a hydrothermal method	N719	I_2_, 1,3-dimethylimidazolium iodide, 4-*tert*-butylpyridine, guanidine thiocyanate, and LiClO_4_ in acetonitrile	3.42%	127
DSSC with 3D TiO_2_ branched nanowire arrays	Sheng *et al.*	Single-crystal-like 3D TiO_2_ branched nanowire arrays consisting of a 1D branch epitaxially grown from the primary trunk prepared by a solvothermal method followed by exposure to O_2_ plasma	N719	1-Hexyl-2,3-dimethylimidazolium iodide and iodine in methoxypropionitrile	4.61%	128
DSSC with TiO_2_ nanowires and TiO_2_ NPs	Bakshayesh *et al.*	Double layered photoanode having corn like TiO_2_ nanowires prepared by a surface tension stress mechanism	N719	Dimethylpropylimidazolium iodide, LiI, I_2_, and 4-*tert*-butylpyridine in acetonitrile	7.11%	129
DSSC with thornbush like TiO_2_ nanowires (TBWs)	Roh *et al.*	Thornbush like TiO_2_ nanowires (TBWs) prepared by a facile single step hydrothermal method	N719	Dimethylpropylimidazolium iodide, LiI, I_2_, and 4-*tert*-butylpyridine in acetonitrile	6.7%	130
DSSC with TiO_2_ nanowire array films	Zhou *et al.*	Vertically oriented, single crystalline rutile TiO_2_ nanowires by a multicycle hydrothermal synthetic process	N719	LiI, I_2_, 1,2-dimethyl-3-*n*-propylimidazolium iodide (DMPII), and *tert*-butylpyridine in dehydrated acetonitrile	2.0%	131
DSSC with TiO_2_ nanowires and TiO_2_ NPs	Zha *et al.*	Double sided brush shaped (DSBS) TiO_2_ nano architecture consisting of highly ordered TiO_2_ nanowires aligned around an annealed TiO_2_ nanoparticle layer was prepared by a hydrothermal method	N719	1-Butyl-3-methyl imidazolium iodide, I_2_, guanidinium thiocyanate, and 4-*tert*-butylpyridine in a mixture of acetonitrile and valeronitrile	5.61%	132
DSSC with TiO_2_ nanowire	Wu *et al.*	Vertically aligned anatase TiO_2_ nanowires on FTO glass with a tunable length in the range of 15–55 mm for multilayered configuration of the photoanode by a hydrothermal method	N719	I^−^/I_3_^−^ redox electrolyte	9.40%	134
DSSC with multistacked three dimensional, hyperbranched titania nanoarchitectures	Wu *et al.*	Photoelectrode with multistacked layers having integrated functions	N719	1-Methyl-3-propylimidazolium iodide (PMII), LiI guanidinium thiocyanate, I_2_, and *tert*-butylpyridine in acetonitrile and valeronitrile	11.01%	135
DSSC with TiO_2_ nanowires	Jiang *et al.*	TiO_2_ nanowire array electrodes are prepared by a hydrothermal method followed by silanization	N719	Ferrocene (Fc), ferrocenium tetrafluoroborate (Fc^+^), and tetrabutylammonium tetrafluoroborate	0.749%	146
DSSC with TiO_2_ nanowires	Li, H. *et al.*	Vertically aligned rutile TiO_2_ nanowire arrays (NWAs) by a single step solvothermal method without using any surfactant or template	C106	DMII, LiI, I_2_, TBP, and GNCS in the mixture of acetonitrile and valeronitrile	8.9%	136
DSSC with a TNW-AuNP hybrid structure	Yen *et al.*	3D TNW-AuNP plasmonic electrode prepared by hydrothermal and sputtering techniques	N719	I_2_, LiI, DMPII, and TBP in acetonitrile	9.73%	137
DSSC with NP/NW composite films	Lee *et al.*	TiO_2_ nanoparticle (NP)/nanowire (NW) composite films with various compositions are prepared by a solvothermal method	MK-2 dye	Cobalt redox couple	7.37%	138
DSSC with a TiO_2_ nanowire network	Jin *et al.*	TiO_2_ nanowire networks on Ti foil using a Ti corrosion reaction	N719	Iodolyte AN-50, Solaronix, Aubonne, Switzerland	1.11%	139
DSSC with TiO_2_ nanowire networks	Shin *et al.*	TiO_2_ nanowire networks on FTO substrates by wet corrosion	N719	Iodolyte AN-50, Solaronix	1.0%	140
DSSC with TiO_2_ nanowires	Li *et al.*	Single crystalline self-branched anatase TiO_2_ NWs by a hydrothermal method using TBAH and CTAB as co-surfactants	N719	LiI, I_2_, dimethylpropylimidazolium iodide (DMPImI) and *tert*-butylpyridine in a dry mixed solution	6.37%	141
DSSC with a Ti@TiO_2_ nanowire array	Liu *et al.*	Spring like Ti@TiO_2_ nanowire array was prepared by a hydrothermal technique at a controlled NaOH concentration	N719	1,3-Dimethylimidazolium iodide I_2_, LiClO_4_, 4-*tert*-butylpyridine and guanidine thiocyanate in acetonitrile	2.787%	142
DSSC with a rutile TiO_2_ nanowire array	Ni *et al.*	Rough surface rutile TiO_2_ nanowire array prepared by a hydrothermal method and prolonged etching. An additional light scattering layer of TiO_2_ particles was also employed	C109	1,3-Dimethylimidazolium, lithium iodide, iodine, *tert*-butylpyridine, and guanidinium thiocyanate in acetonitrile and valeronitrile	9.39%	143
DSSC with reduced graphene encapsulated TiO_2_–B nanowire composite	Makal *et al.*	Reduced graphene oxide encapsulated TiO_2_–B nanowire composite synthesized by a hydrothermal method	N3	Iodolyte AN-50	4.95%	144
DSSC with a g-C_3_N_4_ encapsulated TiO_2_–B nanowire composite	Makal *et al.*	g-C_3_N_4_ encapsulated TiO_2_–B nanowire composite synthesized by thermal calcination of hydrothermally grown TiO_2_–B nanowires and melamine	N3	Iodolyte AN-50	5.12%	145

### 3.2. Nanorods

**Fig. 10:**
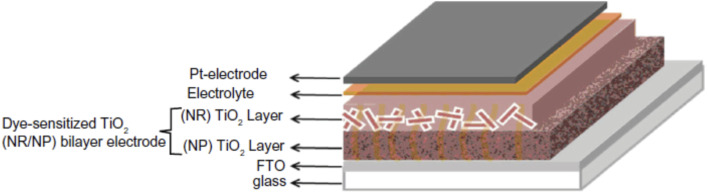
Schematic representation of the DSSC based on a TiO_2_ NR/NP bilayer photoanode.^150^ (Reprinted with permission from ref. Hafez *et al.*, 2010. Copyright 2010 Hafez *et al.*, publisher and licensee Dove Medical Press Ltd.)

**Fig. 11:**
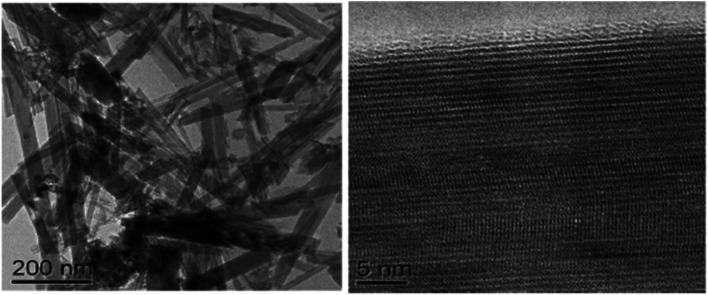
TEM images of the TiO_2_ nanorod sample and the HRTEM image of the nanorod.^150^ (Reprinted with permission from ref. Hafez *et al.*, 2010. Copyright 2010 Hafez *et al.*, publisher and licensee Dove Medical Press Ltd.)

**Scheme 2:**
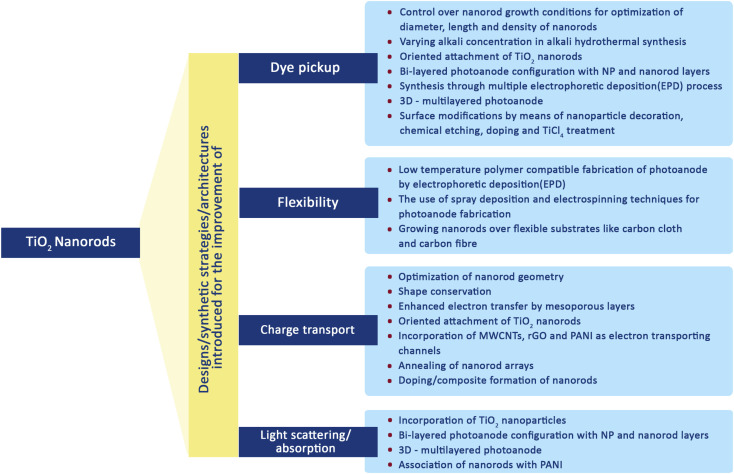
Various modifications introduced for the improvement of the performance of TiO_2_ nanorod based DSSCs.

Nanorods (NRs) are introduced into DSSC photoanode fabrication (Fig. 10) with the objective of increasing DSSC performance by exploiting their one-dimensional nanoscale properties. TiO_2_ nanorods facilitate easy electron transfer by utilising their specific geometry (Fig. 11) and reducing the ohmic loss during the electron transfer through the mesoporous titania layer.^147^ Various methods have been used for the synthesis of TiO_2_ nanorods for DSSC applications. Along with the introduction of novel synthesis strategies, efforts were made to modify the morphology and surface properties of TiO_2_ nanorods as given in Scheme 2.

**Fig. 12:**
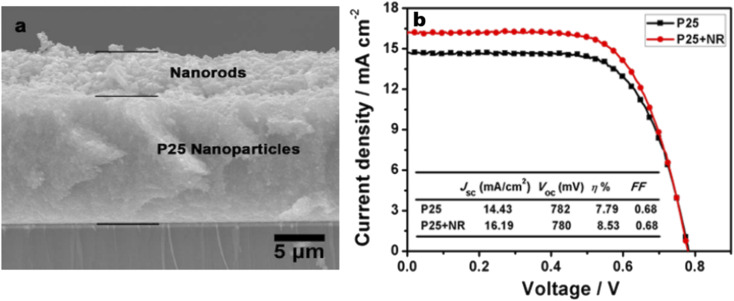
(a) Cross section SEM image of the bi-layer structured photoanode with a P25 nanoparticle bottom layer and porous nanorod top layer; (b) comparison between *J*–*V* curves of cells based on the bi-layer photoanode and single-layer P25 photoanode with the same thickness.^166^ (Reprinted with permission from ref. Chen, H.-Y. *et al.*, 2013.Copyright 2013 American Chemical Society.)

**Fig. 13:**
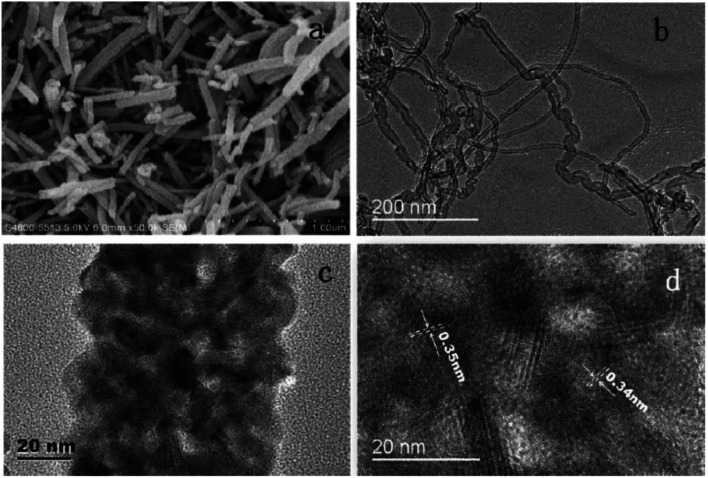
SEM and TEM images: (a) SEM image of TiO_2_ nanorods incorporating MWCNTs; (b) and (c) are TEM images, respectively, of MWCNTs and TiO_2_ nanorods incorporating MWCNTs; (d) HRTEM image of TiO_2_ nanorods that incorporate MWCNTs.^175^ (Reprinted with permission from ref. Yang and Leung, 2013.Copyright 2013 WILEY‐VCH Verlag GmbH & Co. KGaA, Weinheim.)

A widely used fabrication method for TiO_2_ nanorods was the solvothermal method. Several studies on TiO_2_ nanorods were reported based on solvothermal techniques.^147–160^ Most of the solvothermal methods used for TiO_2_ nanorod fabrication employ water as a solvent *i.e.* the hydrothermal method. Sometimes the solvothermal method may be a multi-step process or a combination of more than one synthetic method. In 2005 Jiu *et al.* reported the synthesis of single crystalline anatase TiO_2_ nanorods by a surfactant-assisted hydrothermal method. These nanorods were found to exhibit an efficiency of 7.06%. Higher fill factor (FF) values of TiO_2_ NRs were observed compared to those of P25 nanoparticles, indicating the easier electron transport (lower resistance) through nanorods. In 2006, Jiu *et. al.* prepared single-crystalline anatase TiO_2_ nanorods by a surfactant-assisted hydrothermal method with control over the size and diameter. Here nanorods having a length of 100–300 nm and diameter of 20–30 nm were synthesized and they showed an efficiency of 7.29%.^147^ Jiu *et al.* achieved shape conservation and size regulation of nanorods with the help of a copolymer. Later Liu *et al.* found that the diameter, length and density of the nanorods prepared by the solvothermal method could be varied by changing the growth conditions such as growth time, growth temperature, initial reactant concentration, acidity and additives.^161^ Efforts of Liu *et al.* resulted in the formation of single-crystalline rutile TiO_2_ nanorods having an efficiency of 3%. In the very next year, a significant increase in conversion efficiency (7.9%) was achieved by De Marco *et al.* using TiO_2_ nanorods prepared by a single-step solvothermal technique.^149^ The obtained anatase TiO_2_ nanorod crystals were converted into screen printable paste for easy application into DSSCs. Here the solvothermal method helped to avoid coarse aggregation and shape loss of nanorods. Using the alkali hydrothermal technique, a novel morphology *i.e.* fan-shaped rectangular parallelepiped TiO_2_ rods were synthesized by Shao *et al.* with a conversion efficiency of 6% obtained for 1 M NaOH utilizing DSSCs and this efficiency is found to be 66.7% higher than that of P25-DSSCs.^153,162^ Investigations carried out at different NaOH concentrations showed that the morphology and crystal phase of the nanorods are affected by the alkali concentration. Kathirvel *et al.*, Liu *et al.* and Zhang *et al.* reported the highest efficiencies for solvothermal derived TiO_2_ nanorods.^158–160^ Oriented rutile TiO_2_ nanorod arrays were grown *in situ* on an FTO (fluorine doped tin oxide) coated glass substrate by using a mixed acid medium composed of titanium tetraisopropoxide (TTIP), hydrochloric acid (HCl), acetic acid (AcOH) and water (H_2_O) as the solvent.^152^ An acid mixture with HCl : AcOH in a volume ratio of 4 : 8 produced oriented, uniform, thin rutile nanorods which were found to be superior to single acid-grown nanorods. They have shown an efficiency of 4.03% using 2.3 µm long nanorods. Zhang and co-workers introduced an oriented attachment mechanism for the fabrication of size and shape tuneable anatase TiO_2_ NRs.^158^ These single crystalline long TiO_2_ NRs have reduced grain boundaries which lead to enhanced charge collection. Thus, long thin NR-based DSSCs exhibited an efficiency of 8.87%. In 2015 Liu *et al.* prepared single-crystalline anatase TiO_2_ nanorods by a solvothermal method in which tetrabutylammonium hydroxide (TBAH) is used as the morphology controlling agent.^159^ DSSCs fabricated using these NRs have achieved an efficiency of 8.66%. Recently, several single and multi-step solvothermal fabrications of TiO_2_ NRs were reported.^160,163–165^ Later it is found that employing a composite structure of nanorods (NR) and nanoparticles (NP) in the photoanode can complement the advantages of each other. The first bilayer NR/NP composite structured photoanode was fabricated by Hafez *et al.*^150^ Here the sol–gel derived NP layer was coated with hydrothermally prepared NRs and this DSSC assembly exhibited an efficiency of 7.1%. Several NR/NP bilayered photoanode structures were fabricated later.^151,155,156,166–170^ These bilayer structures provided enhanced light scattering as well as increased surface area, which is finally reflected in their increased efficiencies (Fig. 12). The bilayer design proposed by Rui and co-workers enabled the synthesis of size tuneable rutile TiO_2_ nanorod microspheres by controlled hydrolysis during solvothermal synthesis and can be employed as a scattering over-layer in bilayer DSSCs.^156^ Rui *et al.* achieved an efficiency of 8.22% whereas the efficiency of the single layer reference cell was 7.00%. Better performance of the TiO_2_ NP/NR composite was obtained by Chatterjee and co-workers by employing a 1 : 1 wt% composite obtained from hydrothermally derived NRs and commercially available TiO_2_ powder.^171^ The obtained PCE of 8.61% was the highest among the photoanodes fabricated out of TiO_2_ NR/NP composites. Shao *et al.* introduced low-temperature fabrication of photoanodes for flexible DSSCs by electrophoretic deposition (EPD).^167^ The obtained NR/NP structures (NRPs) have high surface charges and wide size distribution. In addition, a multiple EPD process was adopted to form a better quality microstructured photoanode. In the same total time, the efficiencies of the multiple devices are more than 2.2 times that of one-step devices. Without any calcination or compression, the best device fabricated with multiple EPD and a thin layer of nanoparticles gives a conversion efficiency of 4.35%. Using a two-step hydrothermal method a novel bilayer structure composed of one-dimensional nanorods, which can serve as direct electron transport pathways and a three-dimensional hierarchical structure acting as a light scattering as well as large surface area layer for dye loading was introduced by Li *et al.*^168^ The dependence of the TiO_2_ NR/NP structured composite's photoanode efficiency on the amount of NRs was studied by researchers and it was found that efficiency is the maximum for composites having 10% NR content (4.89%).^172^ Another method employed for nanorod synthesis was electrospinning. Fujihara *et al.* employed electrospun TiO_2_ nanofibres as precursors for nanorods and these rods were spray dried onto an FTO plate.^173^ These spray deposited nanorods overcame the adhesion difficulties of nanofibres and exhibited an efficiency of 5.8%. Another electrospun synthesis was done by Jose *et al.* and they yielded 5.1% efficiency.^190^ In 2009 DSSCs having an efficiency of 9.52% were fabricated by Lee *et al.* using a combination of sol-gel and electrospinning techniques. Here nanorods were electrospun from a solution of titanium *n*-propoxide and polyvinyl acetate in dimethyl formamide.^174^ They have conducted a comparative study of nanorod and nanoparticle based DSSCs. These studies revealed that nanorod DSSCs have a pore volume double that of nanoparticle cells. Obviously the surface area available for sensitizers in nanorods is ∼2.5 times that of the nanoparticle based DSSC at equal TiO_2_ weights. Also, the electron–hole recombination time for nanorods is found to be more than eight times that of nanoparticle DSSCs. Later in 2013 MWCNTs were introduced as electron transporting superhighways into TiO_2_ nanorods by the electrospinning technique (Fig. 13). This incorporation was done by Yang *et al.* and they obtained an efficiency of 10.24%.^175^ In the same year Chen *et al.* employed a microemulsion electrospinning technique to synthesize TiO_2_ nanorods as a composite of mesopores and macropores by using paraffin oil droplets as the template.^166^ This microemulsion electrospinning approach achieved 8.53% efficiency for bilayer DSSC architecture with NRs as the scattering layer.

In 2011 it has been reported that nanorods prepared by the hydrothermal method were used as seeds for the synthesis of a TiO_2_ nano-branched photoanode.^176^ This nano-branched array has achieved an efficiency of 3.75%.

Efforts were made to make NR-based DSSCs flexible by growing NRs on substrates other than the FTO plate. Such an innovative electrode architecture was introduced by Guo and their team, in which bunched TiO_2_ nanorods were grown on carbon fibres by a ‘dissolve and grow’ method.^177^ By using these carbon fibres coated with bunched TiO_2_ nanorods, tube-shaped flexible 3D DSSCs have been prepared with an efficiency of 1.28%. Wang *et al.* made a similar attempt for enhancing flexibility by preparing single crystal nanorod assembled TiO_2_ cloth from carbon cloth templates. Wang *et al.* employed a rapid microwave heating process for TiO_2_ cloth synthesis and this DSSC assembly yielded an efficiency of 2.21%. Wang *et al.* investigated the influence of sensitizers such as N719, C218 and D205 on TiO_2_ nanorod array-based photoanodes and found out that the best efficiency is obtained from C218.^178^

Also, the performance of TiO_2_ NR array DSSCs depends strongly on the annealing temperature.^179,180^ Researchers found that annealed nanorod arrays show an improvement of more than 400% in efficiency compared to unannealed NRs. This increase can be attributed to reduced recombination and better electric contact between the NRs and FTO substrate. Efficiency improvement of TiO_2_ nanorod-based DSSCs can be made by surface modifications such as nanoparticle decoration,^181^ chemical etching,^182^ doping,^183^ TiCl_4_ treatment^184^*etc*. Ghaffari *et al.* showed that the generation of photoelectrons by Au NPs can help electron–hole separation and thereby result in enhanced fill factor (FF) and short circuit current values (*J*_sc_).^181^ The photochemical reduction process under ultraviolet radiation was employed for loading Au NPs on TiO_2_ NRs. Yao *et al.* prepared Nd doped TiO_2_ NRs by the solvothermal method.^183^ Here the doped Nd ions enhanced the injection of excited electrons and thereby decreased the electron–hole recombination. The obtained efficiency was 4.4% which is 33.3% greater than that of the undoped analogue.

Later, a combination of TiO_2_ nanomorphologies with other nanomaterials gained considerable interest. One such photoanode material consists of an overlayer of TiO_2_ nanorods and an underlayer of TiO_2_ embedded ZnO nanoflowers.^185^ The double-layered design was introduced by Chen and co-workers and they achieved a photo conversion efficiency of 8.01%. A similar composite design consists of rutile TiO_2_ nanorods incorporated with an α alumina thin film.^186^ Here α alumina facilitated an enhanced electron lifetime and better charge transport. The α alumina incorporated TiO_2_ nanorod arrays exhibited a PCE of 6.5%.

To boost the charge collection efficiency of TiO_2_ nanorods, a layer of reduced graphene oxide (rGO) was wrapped over the nanorod surface by Subramaniam and his team.^187^ 2 wt% rGO loaded nanocomposite was found to show a superior photo conversion efficiency of 4.54%. Another effort was made by Roy and co-workers to improve the electron transport which involved the wrapping of rutile TiO_2_ nanorod arrays with polyaniline (PANI) on their surface.^188^ Being a conducting polymer, PANI enhanced the electron transport and the conjugation helped to capture more photoelectrons which in turn diminished the recombination between photo excitons. The association of a TiO_2_ NR photoanode with PANI made more visible light absorption possible. Later Tang and co-workers reported that the higher the aspect ratios of TiO_2_ NRs, the higher the dye loading capacity and efficiency will be.^189^ Here NRs having different aspect ratios were prepared by controlling the reaction time. Also, improved performance of branched TiO_2_ NRs was investigated by Hu *et al.*^164^. Prominent research on TiO_2_ nanorod-based DSSC photoanodes is tabulated in Table 2.

**Table 2:** TiO_2_ nanorod-based DSSCs

DSSC Type	Author	Photoelectrode and method of preparation	Sensitizer	Electrolyte	Efficiency	Ref.
DSSC with TiO_2_ nanorods	Jiu *et al.*	TiO_2_ single crystalline anatase nanorods prepared by a surfactant assisted hydrothermal method	N719	LiI, 1,2-dimethyl-3-*n*-propylimidazolium iodide (DMPII), I_2_, and 4-*tert*-butylpyridine (TBP) in methoxyacetonitride	7.06%	148
DSSC with TiO_2_ nanorods	Jiu *et al.*	Highly crystalline TiO_2_ nanorods synthesized by a hydrothermal process in a cetyltrimethylammonium bromide surfactant solution	N719	LiI, 1,2-dimethyl-3-*n*-propylimidazolium iodide (DMPII), I_2_, and 4-*tert*-butylpyridine (TBP) in methoxyacetonitride	7.29%	147
DSSC with TiO_2_ nanorods	Yao *et al.*	Monodispersed Nd-doped TiO_2_ nanorods were synthesized by solvothermal methods	N719	LiI, I_2_, and 4-*tert*-butylpyridine in 3-methoxypropionitrile	4.4%	183
DSSC with TiO_2_ nanorods	Fujihara *et al.*	TiO_2_ nanorods were obtained by mechanical grinding of electrospun TiO_2_ nanofibers	N3 Solaronix	Acetonitrile containing lithium iodide, iodine, 4-*tert*-butylpyridine and 1-propyl-2,3-dimethyl imidazolium iodide	5.8%	173
DSSC with TiO_2_ nanorods	Jose *et al.*	TiO_2_ nanorods by electrospinning	D131, D102, D149 & N3	Acetonitrile containing lithium iodide, iodine, 4-*tert*-butylpyridine, and 1-propyl-2,3-dimethyl imidazolium iodide	5.1%	190
DSSC with TiO_2_ nanorods	Kang *et al.*	TiO_2_ nanorods prepared by an oriented attachment approach	N719	1-Methyl-3-propylimidazolium iodide (MPII), LiI, I_2_, and *tert*-butyl pyridine (TBP) in methoxypropionitrile (MPN)	6.2%	191
DSSC with TiO_2_ nanorods	Lee *et al.*	TiO_2_ nanorod based photoelectrodes prepared by a combination of sol–gel chemistry and electrospinning	N719	1-Butyl-3-methylimidazolium iodide, iodine, guanidinium thiocyanate, and 4-*tert*-butylpyridine in acetonitrile/valeronitrile	9.52%	174
DSSC with TiO_2_ nanorods	Liu *et al.*	Oriented single-crystalline rutile TiO_2_ nanorod films prepared by a hydrothermal method	N719	1-Butyl-3-methylimidazolium iodide, I_2_, *tert*-butylpyridine, and guanidinium thiocyanate in acetonitrile/valeronitrile	3.0%	161
DSSC with TiO_2_ nanorods	De Marco *et al.*	TiO_2_ anatase nanorods prepared by a simple one-step solvothermal method	N719	LiI, I_2_, 1-methyl-3-propylimidazolium iodide, and *tert*-butylpyridine in dried acetonitrile	7.9%	149
DSSC with TiO_2_ nanorods	Hafez *et al.*	TiO_2_ nanorod/nanoparticle (NR/NP) bilayer electrode prepared by a hydrothermal method	N719	Tetrapropylammonium iodide, iodine, lithium iodide, and 4-*tert*-butylpyridine in acetonitrile	7.1%	150
DSSC with a TiO_2_ NP/NR composite	Saji *et al.*	TiO_2_ NPs and NRs were prepared by a surfactant-assisted thermal method	N3	LiI, I_2_, and *tert*-butyl pyridine in 3-methoxy propionitrile	4.89%	172
DSSC with TiO_2_ NRs/NPs	Fan *et al.*	Anatase TiO_2_ fusiform nanorod/NP photoanode prepared by a two step hydrothermal process	N719	LiI, I_2_ and 4-*tert*-butylpyridine in 1 : 1 acetonitrile propylene carbonate	4.68%	151
DSSC with TiO_2_ NRs/NPs	Huang *et al.*	Rutile TiO_2_ nanorod-array films grown from a mixed acid medium by a hydrothermal reaction	(2-Cyano-3-{5′-[1-cyano-2-(1,1,6,6-tetramethyl-10-oxo-2,3,5,6-tetrahydro-1*H*,4*H*,10*H*-11-oxa-3*a*-aza-benzo-[*de*]anthracen-9-yl)-vinyl]-[2,2′]bithiophenyl-5-yl}-acrylic acid	LiI, I_2_, DMPImI, and TBP using dehydrated AN as the solvent	4.03%	152
DSSC with TiO_2_ NRs/NPs	Chatterjee *et al.*	1 : 1 TiO_2_ NR-NP composites prepared by a hydrothermal technique with a hydrogen titanate nanorod precursor	N719	LiI, I_2_ and 4-*t*-butylpyridine in acetonitrile	8.61%	171
DSSC with TiO_2_ nanorod arrays	Shao *et al.*	Fan-shaped rectangular parallelepiped TiO_2_ rods were prepared by an alkali hydrothermal method	N719	LiI, I_2_, and *tert*-butylpyridine in acetonitrile	6.0%	153
DSSC with branched rutile TiO_2_ nanorod arrays	Wang *et al.*	Rutile TiO_2_ nano-branched arrays grown directly on transparent conductive glass (FTO) by a facile two-step wet chemical synthesis process	N719	1,2-Dimethyl-3-propylimidazolium iodide, I_2_, guanidinium thiocyanate, and 4-*tert*-butylpyridine	3.75%	176
DSSC with TiO_2_ nanorod arrays	Wang *et al.*	One dimensional TiO_2_ nanorod arrays prepared by a hydrothermal method	N719, C218 and D205	1-Propyl-3-methylimidiazolium iodide,I_2_, guanidinium thiocyanate, NaI, and *N*-methyl benzimidazole in 3-methoxypropionitrile	1.51%(C218)	178
DSSC with TiO_2_ nanorod cloths	Wang *et al.*	Single-crystal nanorod assembled TiO_2_ cloth with carbon cloth as a template were prepared by a rapid microwave heating process	N719	DMPII, LiI, I_2_ and 4-TBP in methoxypropionitrile	2.21%	192
DSSC with TiO_2_ nanorods	Yang *et al.*	Single crystalline rutile nanorods grown on top of a fluorine doped tin oxide (FTO) substrate *via* a microwave assisted hydrothermal reaction	N719	Iodolyte AN-50, (Solaronix)	3.7%	193
DSSC with a Au NP deposited TiO_2_ nanorod array	Ghaffari *et al.*	Au nanoparticles were loaded onto the surface of hydrothermally prepared TiO_2_ nanorods *via* a photochemical reduction process	N719	Guanidinium thiocyanate (GuSCN), I_2_, 4-*tert*-butylpyridine (TBP) and butylmethylimidazolium iodide (BMII) in a mixture of acetonitrile and valeronitrile	0.94%	181
DSSC with a TiO_2_ nanorod array	Guo *et al.*	Rectangular bunched rutile TiO_2_ nanorod arrays grown on carbon fiber by an acid corrosion method	N719	LiI, I_2_, and 4-*tert*-butylpyridine in 3-methoxypropionitrile	1.28%	177
DSSC with TiO_2_ nanorods	Liu *et al.*	Anatase TiO_2_ nanorods were prepared by a facile two-phase hydrothermal method	N719	LiI, I_2_, 1-methyl- 3-propylimidazolium iodide, and *tert*-butylpyridine in dried acetonitrile	6.37%	154
DSSC with a spherical TiO_2_ nanorod-aggregate light-scattering layer	Liu *et al.*	Bilayer-structured photoelectrode film with spherical TiO_2_ nanorod aggregates as a light-scattering overlayer and nanocrystalline TiO_2_ as an underlayer prepared by a hydrothermal method	N719	LiI, I_2_, 1,2-dimethyl-3-propylimidazolium iodide, and 4-*tert*-butylpyridine in acetonitrile	6.1%	155
DSSC with TiO_2_ nanorods having a composite structure	Chen *et al.*	Ultraporous anatase TiO_2_ nanorods fabricated by a simple microemulsion electrospinning approach	N719	I_2_, LiI, 1-methyl-3-propylimidazolium iodide (PMII), guanidinium thiocyanate, and *tert* butylpyridine in a mixture of acetonitrile/valeronitrile	8.53%	166
DSSC with a spherical TiO_2_ nanorod-aggregate light-scattering layer	Rui *et al.*	TiO_2_ microspheres assembled by single crystalline rutile TiO_2_ nanorods were synthesized by one-pot solvothermal treatment	N719	I_2_, LiI, 1-methyl-3-propylimidazolium iodide (PMII), guanidinium thiocyanate, and *tert*-butylpyridine in a mixture of acetonitrile/valeronitrile	8.22%	156
DSSC with TiO_2_ nanorods and MWCNTs	Yang *et al.*	MWCNTs are introduced into TiO_2_ nanorods by electrospinning	N719	I_2_, LiI, 1-methyl-3-propylimidazolium iodide (PMII) and 4-*tert*-butylpyridine in a mixture of acetonitrile/valeronitrile	10.24%	175
DSSC with a TiO_2_ nanorod based network structure	Yu *et al.*	Three dimensional rutile-nanorod-based network structure was developed by a facile two-step hydrothermal process	N719	Organic-based liquid electrolyte (HPE) that contained a I^−^/I_3_^−^ redox couple was supplied by Dyesol (Australia)	6.31%	157
DSSC with a TiO_2_ nanorod/nanoparticle (NRP) structure	Shao *et al.*	NRP structured photoanodes prepared by electrophoretic deposition (EPD)	N719	LiI, I_2_ and *tert*-butylpyridine in acetonitrile	4.35%	167
DSSC with TiO_2_ nanorods	Zhang *et al.*	Single crystal-like anatase TiO_2_ nanorods with a specific growth direction are prepared by a hydrothermal method	Z907	1,3-Dimethylimidazolium iodide, LiI, and I_2_ in a mixture of acetonitrile and valeronitrile	8.87%	158
DSSC with a bilayered TiO_2_ photoanode	Li *et al.*	Bi-layer photoanode consisting of a hierarchical structure of one dimensional nanorods and three dimensional TiO_2_ was prepared by a hydrothermal method	N719	LiI, I_2_, 1,2-dimethyl-3-propylimidazolium iodide (DMPII) and 4-*tert*-butylpyridine (4-TBP)	5.61%	168
DSSC with TiO_2_ nanorods	Liu *et al.*	Single-crystalline anatase TiO_2_ nanorods were prepared by a solvothermal method	N719	LiI, I_2_,dimethylpropylimidazolium iodide (DMPImI) and *tert*-butylpyridine in dry acetonitrile	8.66%	159
DSSC with TiO_2_ nanorod arrays	Yuan *et al.*	Vertically ordered single-crystalline TiO_2_ nanorod arrays (NRAs) were prepared by a combination of hydrothermal and etching methods	N719	LiI, I_2_, 1-propyl-3-methylimidazolium iodide and *tert*-butylpyridine in dry acetonitrile	4.69%	182
DSSC with TiO_2_ nanorod arrays	Iraj *et al.*	Vertically aligned rutile TiO_2_ nanorod arrays were synthesized by a hydrothermal method	N719	LiI, I_2_, 1-propyl-3-methylimidazolium iodide and *tert*-butylpyridine in dry acetonitrile	1.86%	165
DSSC with TiO_2_ nanorods	Kathirvel *et al.*	Monodispersed TiO_2_ nanorods were prepared using a simple solvothermal process	N719	Lithium iodide, iodine, 4-*tert*-butylpyridine and 1,2-dimethyl-3-propylimidazolium iodide was dissolved in acetonitrile	9.21%	160
DSSC with TiO_2_ nanorods	Tang *et al.*	Single-crystalline anatase TiO_2_ nanorods with a high aspect ratio	N719	LiI, I_2_, dimethylpropylimidazolium iodide (DMPImI) and *tert*-butylpyridine in a dry mixed solution	7.51%	189
DSSC with TiO_2_ nanorods	Guli *et al.*	TiO_2_ nanorod arrays were synthesised through a facile one-step solvothermal route without any surfactant and template	N719		1.68%	163
DSSC with branched hierarchical TiO_2_ nanorod arrays	Hu *et al.*	Rutile branched hierarchical TiO_2_ nanorod arrays were prepared by a facile two-step hydrothermal method	N719	LiI, I_2_, 1-propyl-3-methylimidazolium iodide and *tert*-butylpyridine in dry acetonitrile	2.01	164
DSSC with TiO_2_/ZnO nanoflowers and TiO_2_ nanorod array	Chen *et al.*	Double layered photoanode having an overlayer of a TiO_2_ NR array and underlayer of a TiO_2_ embedded ZnO nanoflower array by a sol–gel method	N719	LiI, I_2_ and LiClO_4_ in acetonitrile	8.01%	185
DSSC with rutile TiO_2_ nanorods incorporated with α alumina	Sriharan *et al.*	Rutile TiO_2_ nanorods incorporated with α alumina were developed on an FTO surface *via* a hydrothermal route	N719	KI, I_2_ and 4-*tert*-butyl pyridine	6.5%	186
DSSC with a 3 dimensional hierarchical TiO_2_ nanorod array wrapped with rGO	Subramaniam *et al.*	Three dimensional hierarchial TiO_2_ nanorod arrays with a layer of rGO wrapping prepared by an *in situ* hydrothermal method	N719	LiI/I_2_ in acetonitrile	4.54%	187
DSSC with PANI wrapped rutile TiO_2_ nanorods	Roy *et al.*	Hydrothermal derived rutile TiO_2_ NRs wrapped with an *in situ* deposited layer of PANI	N719	1-Methylbenzimidazole (NMB) was mixed with a 1 : 1 volume ratio of acetonitrile and 3-methoxypropionitrile (MPN) solution followed by the addition of LiI, tetrabutylammonium iodide (TBAI), and I_2_	4.28%	188

### 3.3. Nanotubes

**Fig. 14:**
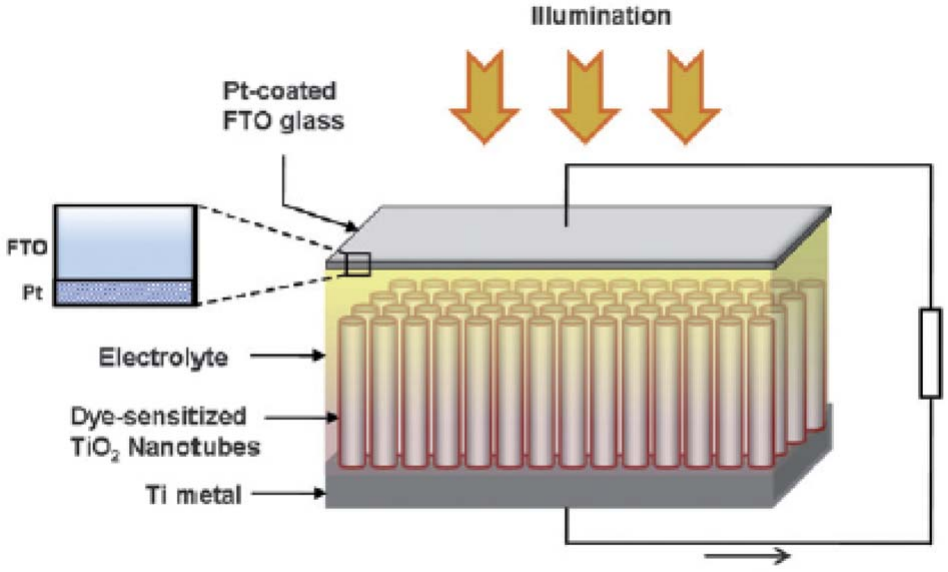
Schematic representation of typical solar cell construction using TiO_2_ nanotubes grown on a Ti substrate.^76^ (Reprinted with permission from ref. Roy *et al.*, 2010. Copyright 2009, Royal Society of Chemistry.)

TiO_2_ nanotubes were introduced with a hollow cavity structure, possessing a higher active surface area. Their enhanced absorption capacity and fast electron transport ability were examined by researchers^194,195^ and they were found suitable for DSSC applications as shown in Fig. 14. Gaps in TiO_2_ mesostructures, which act as electron traps, can be avoided by using nanotube arrays having small boundaries in between. This results in an increased diffusion length *i.e.* the distance travelled by an electron in a tube before recombination.^196^ It was estimated that the diffusion length of a nanotube cell is approximately 100 µm.^197^ So up to this limit, the tube size can be increased which in turn increases the surface area without promoting recombination. The oxidised species from the electrolyte can easily escape from nanotubes because of their ‘open’ structure as compared to from the mesoporous layers. Once it is oxidised, it will diffuse towards the cathode and get reduced back rapidly to minimise recombination losses.^198^ The drawbacks of TiO_2_ nanotubes are their high manufacturing cost and time-consuming preparation techniques.^199^ The efficiency of DSSCs employing TiO_2_ nanotubes is dictated by factors such as morphology and the crystalline structure of the tubes. It is observed that reduction in the tube diameter has a superior role in efficiency compared to the increase in the tube length.^200^ Also, dye loading depends on the annealing temperature of the nanotubes.^200^ The next factor is collection efficiency, which can be improved by minimising recombination losses by introducing modifications on the tube surfaces.^201,202^

**Scheme 3:**
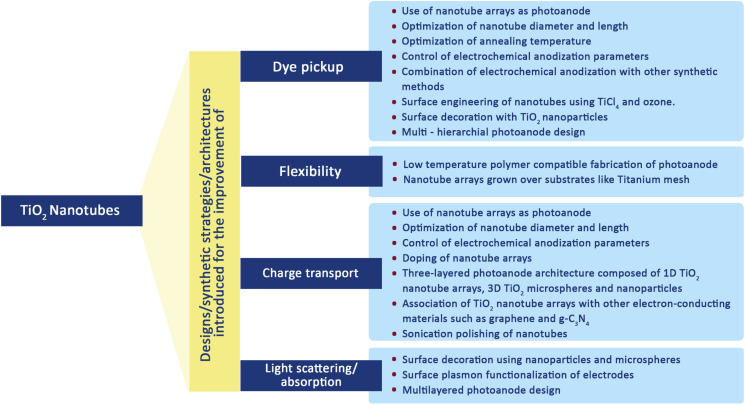
Various modifications introduced for the improvement of TiO_2_ nanotube based DSSC performance.

**Fig. 15:**
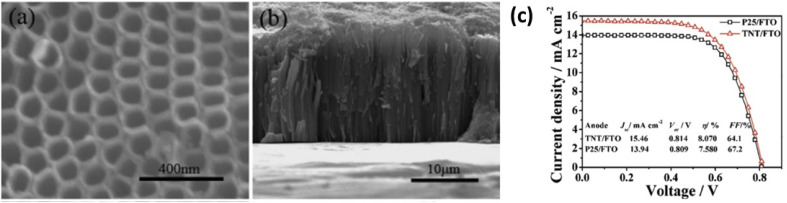
(a and b) FE-SEM images of as-prepared TNT/Ti arrays. (c) Comparison of photovoltaic performance using the same thickness (20.8 µm) of the TNT/FTO film and P25/FTO film under AM 1.5 G illumination (100 mW cm^−2^).^206^ (Reprinted with permission from ref. Lei *et al.*, 2010. Copyright 2010 ECS – American Chemical Society.)

**Fig. 16:**
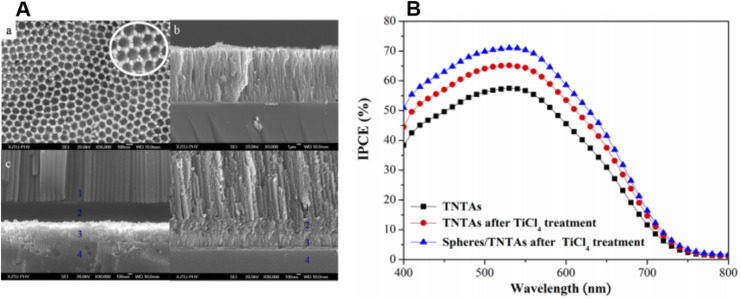
(A) SEM images of highly ordered TiO_2_ nanotube arrays: (a) top view; (b) cross-sectional view; (c) cross-sectional view of the bottom, before TiCl_4_ treatment; and (d) cross-sectional view of the bottom, after TiCl_4_ treatment; (B) incident photon-to-current conversion efficiency (IPCE) curves of solar cells based on TNTAs sensitized by N719.^210^ (Reprinted with permission from ref. He *et al.*, 2013.Copyright 2013, American Chemical Society.)

Scheme 3 provides an overall idea of the efforts made to improve TiO_2_ nanotube based photoanode performance in DSSCs. Some of the significant nanotube-based DSSC architectures are discussed in this section. One of the earliest attempts involved the use of nanotube powders formed by a surfactant template-assisted technique.^203^ These disordered nanotube array has achieved an efficiency of 4.88%.^203^ The most widely employed technique for nanotube synthesis was electrochemical anodization.^202–208^ These electrochemical anodizations were carried out in different electrolytes by different research groups. Yi *et al.* investigated the influence of parameters like water content, anodization time and post-treatment of nanotubes on the photoconversion efficiency of the photoanodes.^209^ It is found that efficient systems can be developed out of a combination of anodization with other synthetic methods for the fabrication of hybrid and multi-layered photoanodes.^210–216^ Another simpler route of TiO_2_ nanotube synthesis proposed by Ramakrishnan and co-workers involved the hydrothermal treatment of quasi-crystalline TiO_2_ nanoparticles.^217^ They were reported to have achieved hydrothermal conditions even without using an autoclave. Apart from using individual nanotubes, designs involving nanotube arrays were made by certain research groups.^194,204,206–208,210,212–215,218–220^ For example, a highly ordered array of TiO_2_ nanotubes with a length of 360 nm was introduced in 2006 by Mor and co-workers with a PCE of 2.9%.^221^ This reduction in PCE is due to the comparatively smaller thickness of the photoelectrode. Mor *et al.* claimed that the efficiency can be further improved towards the ideal limit of ∼31% by increasing the length of the nanotube array up to a few micrometres. Wang *et al.* showed that efficiency can also be increased by treatment of TiO_2_ nanotube arrays with TiCl_4_ and ozone.^202^ Such a surface engineered TiO_2_ nanotube array attained an efficiency of 7.37%. Lei *et al.* brought the efficiency to 8.07%, employing one dimensional TiO_2_ nanotube arrays (Fig. 15a and b) prepared by anodization.^206^ The *I*–*V* plots obtained by Lei and team for the TNT/FTO film in comparison with the P25/FTO film are shown in Fig. 15c. Another report by Hun Park and co-workers shows that TiO_2_ nanotubes longer than 15 µm, transplanted on an FTO plate achieved an efficiency of 5.36% after TiCl_4_ treatment.^207^ TiO_2_ nanotube arrays were also subjected to doping with other elements.^222–224^ The intention of adding dopants is to perfectly match the LUMO of dye molecules to the conduction band of TiO_2_ for efficient electron transfer. This kind of perfect alignment will result in enhanced electron injection and reduced electron recombination. Yang *et al.* introduced TiO_2_ nanotubes doped with Nb by anodizing Ti–Nb alloys (*η* = 3.21%).^222^ Using the same technique, So *et al.* prepared Ru-doped TiO_2_ nanotubes from Ti–Ru alloys(*η* = 5.16%).^223^ While adding metal content it is found that the efficiency is the maximum for an optimum value of metal concentration. Also, metal doping will have an adverse effect if the length of the tubes is not correctly controlled. Subramanian *et al.* fabricated DSSCs with boron-doped TiO_2_ nanotube arrays.^224^ Here B doped nanotube arrays were prepared by anodization and they achieved an efficiency of 3.44% with an increased electron lifetime and reduced interface resistance. Besides doping, surface decoration with NPs and microspheres was also tried by different groups.^205,208,210,214^ Roy *et al.* observed that appropriate treatment of NTAs with TiCl_4_ will result in the decoration of the NTA surface with TiO_2_ nanocrystals.^205^ Such decorations enhanced dye pick up and thereby improved efficiency. He *et al.* decorated a TiO_2_ NTA with TiO_2_ microspheres synthesized by a microwave solvothermal process which yielded an efficiency of 7.24%.^210^Fig. 16 shows SEM images as well as *I*–*V* characteristics of He's photoelectrode. Rho *et al.* introduced open-ended free-standing TiO_2_ NTAs functionalized by Ag NPs and coated by TiO_2_ NPs.^214^ Here nanotube channels were functionalized with Ag NPs to create a plasmonic effect. At the same time, TiO_2_ NPs facilitate scattering. A combination of both these effects resulted in an efficiency of 7.05%. A similar plasmonic enhancement of power conversion efficiency was achieved with the incorporation of Au nanoparticles into TiO_2_ nanotube arrays by Chen *et al.*^225^ The obtained 19% improvement in PCE for the Au nanoparticle inlaid TNT arrays can be attributed to the surface plasmon resonance and scattering effect of the incorporated Au nanoparticles. In the same year, Guo and co-workers came up with a novel single-photon management architecture for a DSSC photoanode.^226^ Their design, consisting of a TiO_2_ nanotube photonic crystal deposited with Au nanoparticles, achieved a PCE of 5.63% which was the result of the synergistic effect of a photonic crystal and surface plasmon resonance of Au nanoparticles. Followed by this, in 2019, Fu *et al.* developed a cigar-like Au/TiO_2_ nanotube array/TiO_2_ nanoparticle multi-hierarchical photoanode with a PCE of 8.93%.^227^ The multi-hierarchical design, which was obtained *via* a novel vacuum-assisted colloid filling route has 4 times better charge transport and 3.2 times better dye intake than that of the conventional TiO_2_ nanotube array-based photoanodes. Cirak and co-workers proposed a hybrid photoanode design comprising ZnO nanorods and TiO_2_ nanotubes.^228^ The hybrid photoanode fabrication was carried out by a two-step synthetic process, anodic oxidation of TiO_2_ nanotubes followed by hydrothermal deposition of ZnO nanorods over the TiO_2_ nanotubes. They found the hydrothermal treatment temperature to be a determining factor of PCE. The hybrid design attained a PCE almost double that of the normal TiO_2_ nanotube photoanode. As part of achieving increased dye pick up and enhanced light scattering, multi-layered photoanode architectures involving TiO_2_ nanotubes were also introduced.^208,210,211,213,214,216^ As mentioned in the case of nanowires and nanorods (Sections 1.8 and 1.9), here also specific functions were assigned to individual layers. In 2014 Wu *et al.* introduced a three-layered photoanode architecture composed of 1D TiO_2_ nanotube arrays, 3D TiO_2_ microspheres and zero dimensional nanoparticles.^211^ This novel structure has inherent properties such as high dye loading capability, efficient light scattering capacity and enhanced charge collection ability, leading to an unprecedented increase in photoconversion efficiency *i.e.,* 9.10%. Later several other research groups fabricated such tri-layered photoanodes with various modifications.^213,216^

Several other associations of TiO_2_ nanotube arrays with other electron-conducting materials such as graphene and g-C_3_N_4_ were later introduced. Wang *et al.* fabricated a single-layer graphene/TiO_2_ nanotube array heterojunction photoanode by a wet transfer method and achieved a photon to current conversion efficiency of 4.18% which is far higher than that of pristine TiO_2_ nanotube photoanodes.^229^ The formation of a heterojunction with graphene not only reduced the bandgap but also enhanced visible light absorption. Mohammadi and co-workers modified TiO_2_ nanotube arrays with g-C_3_N_4_ and ZnO nanoparticles through a hydrothermal route. Even though there is no significant improvement in PCE by this composite formation, the *I*_sc_ and *V*_oc_ of the hybrid photoanode showed considerable improvement. In addition to the above-mentioned investigations, some distinct approaches were also attempted by researchers. For example, Lee *et al.* investigated the influence of various types of glass substrates on the performance of DSSCs fabricated from TiO_2_ nanotubes.^230^ They found that the photoconversion efficiency shows a significant increase with an increase in the conductivity of the glass used. Apart from the conventional cylindrical-shaped nanotubes, So *et al.* fabricated cone-shaped TiO_2_ nanotube arrays with improved photon management capability.^231^ When used in back side illuminated DSSCs, nanocone structures exhibited efficiencies near 8% which is the highest efficiency reported for this type of DSSC. Later in 2014, open-ended TiO_2_ nanotubes prepared by electrochemical anodization of Ti foil were used for DSSC fabrication by Hou *et al.* and these DSSCs were subjected to stability studies by *I*–*V* characterisation.^220^ Their results indicate that the PCE decay of the DSSCs depends on the *J*_sc_, instead of the *V*_oc_ and FF, with the extension of testing time. The stability of DSSCs mainly depends on the sealing properties and the volatile electrolyte of the devices. In 2016 Gu *et al.* reported the removal of the capping layer over the TiO_2_ nanotubes and subsequent enhancement of charge transport by sonication polishing treatment.^212^ This sonication polishing helped the removal of flocculent substances atop TiO_2_ nanotube arrays (NTAs) and also smoothened their outer surface. They have obtained an efficiency of 6.28%. Zhu *et al.* synthesized open-ended TiO_2_ nanotube arrays by removing the closed bottom caps by ball milling.^232^ The fast ball removal of bottom caps enhanced their surface area and the open-ended photoanode exhibited a PCE of 7.7%. As a generalisation, the common modification strategies of TiO_2_ nanotubes include (a) attaching light-harvesting molecules *i.e.* sensitization of nanotubes,^206,215^ (b) doping,^218,222–224,233^ (c) band gap engineering,^202^ (d) inducing electronic heterojunctions^204,219,234^ and (e) secondary semiconductor particle decoration.^205^ An overview of DSSC applications of the tubular morphology of TiO_2_ nanomaterials is given in Table 3.

**Table 3:** TiO_2_ nanotube based DSSCs

DSSC Type	Author	Photoelectrode and method of preparation	Sensitizer	Electrolyte	Efficiency	Ref.
DSSC with TiO_2_ nanotubes	Adachi *et al.*	Titania nanotubes were synthesized using molecular assemblies	N3	0.03 M iodine in 0.3 M lithium iodide in 3-methyl-2-oxazolidinone (NMO)/acetonitrile	4.88%	203
DSSC with a TiO_2_ nanotube array	Maggie *et al.*	Highly ordered TiO_2_ nanotube arrays are made by anodization of a titanium film in a fluoride containing electrolyte	N719	LiI, I_2_, *N*-methylbenzimidazole, guanidinium thiocyanate, and *tert*-butylpyridine in methoxypropionitrile (MPN)	4.7%	204
DSSC with TiO_2_ nanotubes decorated with TiO_2_ NPs	Roy *et al.*	TiO_2_ nanotubes were prepared by anodization of Ti foil followed by annealing and TiCl_4_ treatment	N719	Iodolyte R50 (Solaronix)	3.8%	205
DSSC with a TiO_2_ nanotube array	Wang *et al.*	Highly ordered TiO_2_ nanotube arrays were fabricated by electrochemical anodization followed by surface engineering using TiCl_4_ and O_2_ plasma	N719	BMIM-I, I_2_, TBP, and GTC in acetonitrile/valeronitrile	7.37%	202
DSSC with a ZnO nanorod and TiO_2_ nanotube hybrid photoanode	Cirak *et al.*	Hybrid photoanode with ZnO nanorods and TiO_2_ nanotubes prepared through anodic oxidation followed by hydrothermal deposition	N719	I^−^/I_3_^−^ redox pair (Hi-30, Solaronix)	1.67%	228
DSSC with a TiO_2_ nanotube array	Lei *et al.*	Highly ordered one-dimensional TiO_2_ nanotube arrays were prepared by anodization of pieces of Ti foil	N719	I_2_, 1-methyl-3-propylimidazolium iodide (PMII), guanidinium thiocyanate, and *tert*-butylpyridine in acetonitrile and valeronitrile	8.07%	206
DSSC with TiO_2_ nanotube arrays	Park *et al.*	Highly ordered TiO_2_ nanotube arrays were fabricated by anodization and transplanted on to an FTO substrate	N719	1-Hexyl-2,3-dimethyl-imidazolium iodide (C6DMIm), iodine (I_2_), lithium iodide (LiI), and 4-*tert*-butylpyridine in 3-methoxypropionitrile	5.36%	207
DSSC with Nb doped TiO_2_ nanotube layers	Yang *et al.*	Nb-doped TiO_2_ nanotube layers were prepared by electrochemical anodization of Ti–Nb alloys	N719	I_3_^−^/I^−^ as the redox electrolyte	3.21%	222
DSSC with a TiO_2_ nanotube array/NPs	Zheng *et al.*	Layer-by-layer assembly of self-standing titania nanotube arrays and NPs were prepared by anodization	Ru535-bisTBA	1,2-dimethyl-3-propyl imidazolium iodide, LiI, I_2_, and 4-*tert*-butylpyridine in acetonitrile	8.80%	208
DSSC with Ru doped TiO_2_ nanotubes	So *et al.*	Ru doped TiO_2_ nanotubes were prepared by anodization of Ti–Ru alloys	D-719, (Eversolar, Taiwan)	Iodolyte R50 (Solaronix)	5.16%	223
DSSC with B doped TiO_2_ nanotube arrays (TNAs)	Subramanian *et al.*	B doped TiO_2_ nanotubes were prepared by anodization of Ti foil in the presence of 0.5 wt% NH_4_F and 5 wt% 0.5 M boric acid in ethylene glycol solution	N719	Iodolyte R-150	3.44%	224
DSSC with TiO_2_ nanotubes and TiO_2_ microspheres	He *et al.*	TNTAs were prepared by anodization of pieces of Ti foil in an aqueous solution containing hydrofluoric acid and TiO_2_ scattering microspheres were prepared *via* a microwave solvothermal process	N719	I_2_, 1-methyl-3-propylimidazolium iodide (PMII), guanidinium thiocyanate, and *tert*-butylpyridine in a solution of valeronitrile and acetonitrile	7.24%	210
DSSC with a TiO_2_ nanotube photoelectrode and transparent Pt counter electrode	Lee *et al.*	Ti layers deposited over an FTO substrate were completely anodized to obtain TiO_2_ nanotubes	D-719, Eversolar, Taiwan	Iolilyt SB-163(Iolitec, Germany)	7.58%	230
DSSC with TiO_2_ nanotube/microsphere/NPs tri-layered photoanode	Wu *et al.*	Double layered photoanode with a 1D NT underlayer and 3D hierarchical upper layer was prepared by a hydrothermal method and then incorporated with hydrothermally prepared TiO_2_ NPs	N719	1-Methyl-3-propylimidazo-lium iodide (PMII), guanidinium thiocyanate, I_2_, LiI, and *tert*-butylpyridine dissolved in acetonitrile and valeronitrile	9.10%	211
DSSC with an open ended TiO_2_ nanotube array	Hou *et al.*	Vertically oriented, high-aspect-ratio TNT arrays were prepared by two-step anodic oxidation	N719	1-Methyl-3-propylimidazo-lium iodide (PMII), guanidinium thiocyanate, I_2_, LiI, and *tert*-butylpyridine dissolved in acetonitrile and valeronitrile	5.01%	220
DSSC with cone shaped TiO_2_ nanotube arrays	So *et al.*	Cone shaped TiO_2_ nanotubes were prepared by anodization followed by TiCl_4_ treatment	N719	BMIM-I, I_2_, and GTC in acetonitrile/valeronitrile (SB-163, IoLiTec Inc, Germany)	8%	231
DSSC with TiO_2_ nanotube arrays	Gu *et al.*	TiO_2_ NTAs were synthesized by a modified electrochemical anodic oxidation method *i.e.* sonication followed by anodization. Then the nanotubes were filled with Sb_2_S_3_ by chemical bath deposition	N719	LiI, I_2_, and *tert* butylpyridine in acetonitrile	6.28%	212
DSSC with a composite of P25 NP/TiO_2_ NTA/flower like TiO_2_	Hu *et al.*	TiO_2_ NTAs prepared by anodization were transplanted on to a P25 coated FTO plate and then flower like TiO_2_ prepared hydrothermally was deposited over a P25/NTA film	N719	LiI, I_2_, 4-*tert*-butylpyridine and 1, 2-dimethyl-3-propylimidazolium iodide (DMPII) in dry acetonitrile	6.48%	213
DSSC with Ag nanoparticle-Functionalized open-ended freestanding TiO_2_ nanotube arrays	Rho *et al.*	TiO_2_ nanotube arrays prepared by anodization, with channels containing Ag NPs coated with TiO_2_ NPs for light scattering	N719	I_2_, guanidium thiocyanate (GSCN), and 4-*tert*-butylpyridine (TBP) in a mixture of acetonitrile and valeronitrile	7.05%	214
DSSC with a Au/TiO_2_ nanotube array/TiO_2_ nanoparticle photoanode	Fu *et al.*	“Cigar-like” Au/TiO_2_-nanotube-array/TiO_2_-nanoparticle multi-hierarchical photoanode through a novel vacuum-assisted colloid-filling approach	N719	LiI, I_2_, 1-methyl-3-hexylimidazolium iodide (HMII), *N*-methylbenzimidazole (NMB) and 4-*tert*-butylpyridine in 3-ethoxypropionitrile	8.93%	227
DSSC with TiO_2_ nanotube arrays	Peighambardoust *et al.*	Pieces of Ti foil were degreased ultrasonically and then anodized to form TiO_2_ NTAs	N719	Ionic-salt-based liquid electrolyte	3.14%	215
DSSC with a tri-layered photoanode	Wu *et al.*	Tri-layered photoanode consisting of single crystal hollow TiO_2_ nanoparticles (HTNPs), sub-micro hollow TiO_2_ mesospheres (SHTMSs) and hierarchical TiO_2_ microspheres (HTMSs)	N719	1-Methyl-3-propylimidazo-lium iodide (PMII), guanidinium thiocyanate, I_2_, LiI, and *tert*-butylpyridine dissolved in acetonitrile and valeronitrile	9.24%	216
DSSC with an open ended TiO_2_ nanotube array photoanode	Zhu *et al.*	Open ended TiO_2_ nanotube array photoanode was prepared by fast removal of bottom caps by mechanical ball milling	N719	LiI, I_2_, 1-methyl-3- hexylimidazolium iodide (HMII), *N*-methylbenzimidazole (NMB), and 4-*tert*-butylpyridine in 3-methoxypropionitrile	7.7%	232
DSSC with a single layer graphene–TiO_2_ nanotube array heterojunction	Wang *et al.*	Single layer graphene–TiO_2_ nanotube array heterojunction photoanode synthesized by a wet transfer method	N719	Ethylene glycol solution containing NH_4_F and H_2_O	4.18%	229

### 3.4. Effect of one-dimensional nanomaterial morphology on electronic and charge transport properties

Compared to bulk TiO_2_ having a wide bandgap, one dimensional TiO_2_ nanostructures exhibited enhanced surface area, more active sites and quantum confinement effects. Size, specific crystal facets, morphology and alignment are the main factors determining the electronic properties of these one – dimensional TiO_2_ structures. In the case of nanowires, as the size reduces to the nanoscale regime, the bandgap starts increasing as per quantum confinement.^66^ Doping of foreign elements is the main strategy employed for the tuning of the bandgap and such dopants can align the valence band and conduction band positions in favour of the sensitizer energy levels. Also, the best DSSC performances were obtained for vertically aligned nanowire based photoanodes. In the case of nanorods, significant reduction in grain boundaries which act as electron traps caused an increment in the electron diffusion length and thereby enhanced charge transfer.^174,178^ The bandgap adjustment can be made in TiO_2_ nanorods by varying the aspect ratio of nanorods. With an increase in the aspect ratio of nanorods, there will be a downshift of the conduction band edge.^66^ Similarly, surface area enhancement can be achieved by introducing surface roughness and mesoporosity.^235^ In the case of TiO_2_ nanotubes, those having a wall thickness less than 5 nm are capable of manifesting quantum confinement effects.^236^ Only hydrothermal tubes fall under this category. The presence of Ti^3+^ and oxygen vacancies are another reason for enhanced charge transport. Annealing temperature is a crucial factor determining the charge transport ability of nanotubes.^236^ Bandgap engineering in nanotubes can be made by doping, surface adsorption, heterojunction formation and surface decoration.^76,205,222,223^

### 3.5. Significance of one-dimensional TiO_2_ nanomorphology for exploitation of the photoanode performance

TiO_2_ one-dimensional (1D) morphologies were introduced as photoanode materials to overcome the limitations encountered by TiO_2_ nanoparticulate electrodes. These one-dimensional morphologies are found to have profound photoelectrode characteristics with certain constraints which limit their use as photoanodes. We address this problem to highlight a particular one-dimensional morphology as the best choice by analysing various TiO_2_ nanomaterials with 1D morphologies. A clear distinction of the best among nanowires, nanorods and nanotubes as photoanode materials requires a detailed investigation of several aspects. The main aspects to be considered are the band gap, surface area, aspect ratio, charge recombination time, transport properties and stability. In terms of surface area, nanowires, nanorods and nanotubes are inferior to zero-dimensional TiO_2_ nanoparticles.^89^ However, they possess high aspect ratios and better charge transport pathways compared to conventional multidimensional TiO_2_ NPs. As a result of limited surface area, the amount of dye (sensitizer) intake into these one-dimensional morphologies would be lesser. Even then, DSSCs based on TiO_2_ one-dimensional photoanodes exhibit better IPCEs. It was found that the TiO_2_ NP layer, having thrice the surface area of a TiO_2_ nanotube layer, exhibited a much lower IPCE compared to the nanotube layer.^237^ This enhancement in IPCE of nanotubes can be attributed to the much larger electron transport time prevailing in them.^237^ In the case of nanotube photoanode materials, the fraction of dye intake as well as electron diffusion lengths increases with an increase in the tube length. The electron diffusion lengths in nanotubes are almost thirty times that of NPs.^236,237^ Thus, an increased tube length can have the synergic effect of both surface area enhancement and smoother electron transport, irrespective of the high density of electron trap states in TiO_2_ nanotubes. Similar results are obtained in the case of TiO_2_ nanowires also. The best efficiencies are obtained upon arranging the nanowires vertically rather than randomly, which ensures effective contact between individual nanowires and the electrode.^66^ With an increase in the length of nanowires, the number of electron traps and dye loading increases. So, finding the optimal nanowire length ensures the best IPCE. The TiO_2_ nanorod-based photoanode exhibited an electron lifetime 8 times that of a TiO_2_ NP-based one.^174^ TiO_2_ nanorods showed increased electron diffusion lengths and reduced grain boundaries compared to NP-based electrodes.^191^ The above information leads us to the conclusion that the selection of the best one-dimensional TiO_2_ photoanode material among the three *i.e.*, nanorods, nanowires and nanotubes is rather difficult. This dilemma arises due to the vast number of modifications possible over one dimensional TiO_2_ nanomaterials using various design strategies under different experimental conditions. Several of these investigations have led to significant and valuable findings regarding the photoanode architecture, performance parameters and stability. Upon a detailed survey of the literature, it can be seen that the combination of one dimensional TiO_2_ nanomaterials with other nanomaterials exhibited the best conversion efficiencies. These nanomaterials are chosen in such a way that the limitations of one-dimensional TiO_2_ morphologies can be made up by them. Particle decoration, heterojunction formations and multi-layered architectures are superior design strategies employed for the fabrication of modified one-dimensional TiO_2_ photoanodes. The limited surface area of the one-dimensional structures is improved by the addition of TiO_2_ NPs. These added layers cause enhanced light scattering and better dye loading capability. Decoration with metal nanoparticles makes use of surface plasmon resonance and yields better light absorption and electron transport. Heterostructures of one-dimensional TiO_2_ with materials having suitable band structures and electron transport properties exhibit improved efficiency. From the above literature survey (Tables 1, 2 and 3), it can be seen that top IPCE values were obtained for multi-layered photoanodes involving one-dimensional TiO_2_ morphologies. Each layer was incorporated with a specific function to be carried out, and such a division of labour led to enhanced outputs. Single layered one-dimensional TiO_2_ morphologies prepared by advanced techniques like electrospinning, anodization, *etc.* or a combination of these techniques with sol-gel and hydrothermal methods yield higher efficiencies.

## 4. Summary and future prospects

This review discusses the design and working principle of various TiO_2_ nanomorphologies and surveys various aspects of TiO_2_ based photoanode materials in dye-sensitized solar cells. The total reliance of mankind on non-renewable energy sources has resulted in their depletion as well as serious environmental pollution. In this scenario, sunlight is considered a universal source of energy which is abundant in nature and free of cost. Thus, solar energy and its conversion into electricity and fuel (hydrogen) can be exploited. Solar cells which convert sunlight into electricity can be considered a future remedy for the energy crisis. In recent years a wide variety of TiO_2_ nanostructures have been synthesized as photoanodes for DSSC applications such as nanoparticles, nanowires, nanorods, nanotubes, *etc.* Due to their excellent scattering ability and high electron transport rate, these one-dimensional nanostructures are found to be efficient candidates for DSSC photoanode fabrication. By overcoming their limitations with surface area, even higher efficiencies can be achieved. The suitable band alignment of TiO_2_ with the sensitizer and the subsequent charge transport properties make it more attractive among other semiconductors in addition to its non-toxicity and viable functional architecture. TiO_2_ nanomaterials have a band gap in the UV region of solar spectra which restricts their use in the field of visible active applications. In DSSCs, the electrons in the LUMO of the dye are transferred to the conduction band of TiO_2_ nanomaterials. Anion and cation doping in TiO_2_ nanomaterials are remarkable routes to make suitable alignments of the LUMO of the dye and conduction band position of TiO_2_ for fast electron transfer. Recent reports show that the IR absorption capability of TiO_2_ can be enhanced by extending its absorption edge into the infra-red region which provides a high charge collection.^116,238^

## Conflicts of interest

There are no conflicts to declare.

## Supplementary Material
